# Tissue glycomics distinguish tumour sites in women with advanced serous adenocarcinoma

**DOI:** 10.1002/1878-0261.12134

**Published:** 2017-09-29

**Authors:** Merrina Anugraham, Francis Jacob, Arun V. Everest‐Dass, Andreas Schoetzau, Sheri Nixdorf, Neville F. Hacker, Daniel Fink, Viola Heinzelmann‐Schwarz, Nicolle H. Packer

**Affiliations:** ^1^ Department of Chemistry & Biomolecular Sciences Biomolecular Discovery & Design Research Centre Faculty of Science and Engineering Macquarie University North Ryde NSW Australia; ^2^ Glyco‐oncology Ovarian Cancer Research Department of Biomedicine University Hospital Basel University of Basel Switzerland; ^3^ Australian Research Council Centre of Excellence in Nanoscale Biophotonics Macquarie University North Ryde NSW Australia; ^4^ Glycomics Institute Griffith University Gold Coast Queensland Australia; ^5^ Ovarian Cancer Research Department of Biomedicine University Hospital Basel University of Basel Switzerland; ^6^ Gynecological Research Adult Cancer Program Lowy Cancer Research Centre University of New South Wales Sydney Australia; ^7^ Royal Hospital for Women Gynecological Cancer Centre School of Women's and Children's Health University of New South Wales Sydney Australia; ^8^ Department of Gynecology University Hospital Zurich Switzerland; ^9^ Hospital for Women Department of Gynecology and Gynecological Oncology University Hospital Basel University of Basel Switzerland

**Keywords:** gene expression, glycans, mass spectrometry, ovarian cancer, peritoneal cancer, porous graphitized carbon

## Abstract

In the era of precision medicine, the tailoring of cancer treatment is increasingly important as we transition from organ‐based diagnosis towards a more comprehensive and patient‐centric molecular diagnosis. This is particularly the case for high‐grade serous adenocarcinomas of the ovary and peritoneum, which are commonly diagnosed at an advanced stage, and collectively treated and managed similarly. We characterized the *N*‐ and *O*‐glycome of serous ovarian (OC) and peritoneal cancer (PC) tissues using PGC‐LC‐ESI‐IT‐MS/MS profiling and validated the discriminatory glycans and their corresponding glyco‐gene expression levels using cell lines and transcriptomic data from 232 patients. Overall, the *N*‐ and *O*‐glycan repertoires of both cancer types were found to comprise mostly of α2,6‐sialylated glycan structures, with the majority of *N*‐glycans displaying the biantennary mono‐ and disialylation as well as bisecting‐type biantennary glycans. The MS profiling by PGC‐LC also revealed several glycan structural isomers that corresponded to LacdiNAc‐type (GalNAcβ1‐4GlcNAc) motifs that were unique to the serous ovarian cancers and that correlated with elevated gene expression of *B4GALNT3* and *B4GALNT4* in patients with serous cancer. Statistical evaluation of the discriminatory glycans also revealed 13 *N*‐ and 3 *O*‐glycans (*P *<* *0.05) that significantly discriminated tumour‐sampling sites, with LacdiNAc‐type *N*‐glycans (*m*/*z* 1205.0^2−^ and *m*/*z* 1059.4^2−^) being associated with ovarian‐derived cancer tissue and bisecting GlcNAc‐type (*m*/*z* 994.9^2−^) and branched *N*‐glycans (*m*/*z* 1294.0^2−^ and *m*/*z* 1148.4^2−^) upregulated at the metastatic sites. Hence, we demonstrate for the first time that OC and PC display distinct molecular signatures at both their glycomic and transcriptomic levels. These signatures may have potential utility for the development of accurate diagnosis and personalized treatments.

**Abbreviations**
BPC: base peak chromatogram
EIC: extracted ion chromatogram
ESI: electrospray ionization
FIGO: International Federation of Gynecology and Obstetrics
Fuc: l‐fucose
Gal: d‐galactose
GalNAc: *N*‐acetyl‐d‐galactosamine
Glc: d‐glucose
GlcNAc: *N*‐acetyl‐d‐glucosamine
GLM: generalized linear model
HGSC: high‐grade serous cancer
IT: ion trap
LacdiNAc: *N*,*N*′‐diacetyl‐lactosamine
LC: liquid chromatography
Man: d‐mannose
MS: mass spectrometry
Neu5Ac: *N*‐acetyl‐neuraminic acid
OC: high‐grade serous ovarian cancer
PC: high‐grade serous peritoneal cancer
PGC: porous graphitized carbon
RF: random forest

## 1 Introduction

Epithelial ovarian cancer has the highest mortality rate of all gynaecological malignancies, because more than 75% of tumours are diagnosed at an advanced FIGO (International Federation of Gynecology and Obstetrics) stage (III and IV) (Coleman *et al*., 2011) where the 5‐year survival rate is only 20% (Fishman and Bozorgi, 2002; Ozols, 2006). Compared to many other solid cancer types, the prognosis for ovarian cancer has changed little since platinum‐based treatment was introduced more than 30 years ago (Vaughan *et al*., 2011). Epithelial ovarian cancer is a heterogeneous group of cancers, classified primarily on their histopathological features (serous being the most common subtype) and location of the primary tumour mass (Kurman and Shih Ie, 2010; Scully *et al*., 1998). Serous ovarian and peritoneal tumours are believed to be frequently derived from the fallopian tube, may not be distinguishable preoperatively (Barda *et al*., 2004), display a similar genetic predisposition (*BRCA* mutations) (Bandera *et al*., 1998a,b) and commonly present with widespread dissemination within the pelvis and abdomen (Kessler *et al*., 2013; Seidman *et al*., 2011). Despite being treated identically, there is a debate as to whether these cancers are variants of the same malignancy or develop through different pathways. Difficulties in the identification of the site of origin in patients with advanced disease, where the ovary, fallopian tube and the abdominal cavity are all usually involved, plus their macroscopic and microscopic resemblance, are major causes of this debate.

Recent findings suggest that peritoneal and ovarian cancer may be linked to different carcinogenic pathways, with both cancers exhibiting differences in protein expression of Her2/neu, estrogen and progesterone receptors (Sorensen *et al*., 2015). This is in line with elevated plasma levels of the routinely used clinical biomarkers, HE4 and CA125, in peritoneal compared to ovarian and tubal cancers (Jacob *et al*., 2011). There is also increasingly epidemiological evidence that peritoneal cancer and ovarian cancer are two distinct diseases (Gao *et al*., 2016; Rottmann *et al*., 2017; Schnack *et al*., 2014). It is evident that studies investigating the underlying differences within these serous cancer subtypes which are reflective of the individual cellular phenotypes and molecular origin have been rather limited (Chen *et al*., 2003; Lacy *et al*., 1995; Pere *et al*., 1998). It has also been shown that a majority of fallopian tubes examined from patients with primary peritoneal cancer have been found with the presence of serous tubal intraepithelial lesions, thereby giving the tumour cells access to the peritoneal cavity (Seidman *et al*., 2011). Despite the use of high‐throughput ‘omics’ technologies that are rapidly increasing, no studies have elucidated the characteristic molecular profiles of these morphologically related cancer tissue samples within this context. Specifically, the degree to which these serous tumours differ, despite their similarity in terms of pathogenesis, clinical behaviour and chemotherapy response, remains to be elucidated.

It has been widely established that protein glycosylation is an important post‐translational modification, which has relevance in many biological processes such as cell signalling, immune responses, extracellular interaction and cell adhesion (Cummings and Pierce, 2014). In fact, several studies have elucidated a plethora of cellular‐ and *in vivo* tissue‐specific glycan patterns on glycoproteins, thereby providing an insight into the specificity of glycosylation activities (Chandrasekaran *et al*., 2006; Sun *et al*., 2011). In the tumour microenvironment, aberrant glycosylation has been well documented, with increasing evidence pointing to the role of *N*‐ and *O*‐glycans in modulating critical aspects of tumour growth and development as well as metastasis. These structures resemble molecular‐level glycomic ‘fingerprints’ which facilitate the discrimination between healthy and diseased states or reflect tumour microheterogeneity caused by the variation within cancer subtypes (Abbott *et al*., 2010; Christiansen *et al*., 2014; West *et al*., 2010). While several studies yielded clinically relevant information on the differential expression of ovarian cancer‐associated glycans found in patients’ serum and tissues, the major focus of their analysis was on differentiating between healthy and diseased patients (Abbott *et al*., 2008; Jacob *et al*., 2012, 2014a). In regard to cell surface glycosylation, there is still a lack of studies that have utilized glycomics to characterize the specific *N*‐ and *O*‐glycan structures within the serous‐histotype groups of tumours which likely represent distinct diseases despite still being classified as ovarian cancer.

To address this challenge, we have employed a quantitative MS‐based glycomics profiling of plasma membrane *N*‐ and *O*‐glycoproteins derived from invasive adenocarcinomas of the ovary and peritoneum. We also sought to identify specific glycotopic features of these serous cancer tissues using porous graphitized carbon (PGC) chromatographic retention and mass spectrometric fragmentation characteristics, and to validate the presence of discriminatory glycans using a combination of statistical classification methods and glyco‐gene transcriptomic data derived from an independent cohort. This comprehensive tissue glycan profiling serves as the first step towards identifying serous cancer glycan biomarkers that may be utilized in diagnostics or further applied to various MS imaging technology platforms for the robust and sensitive discrimination of tumours.

## 2 Results

**Figure 1:**
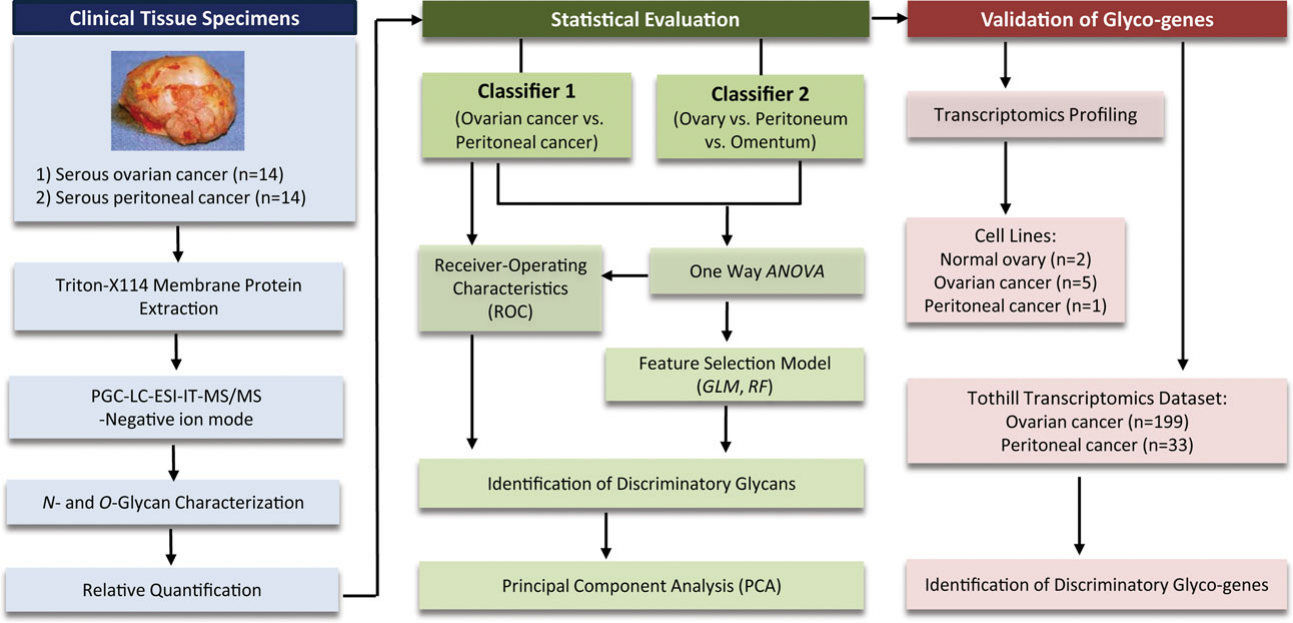
Graphic illustration of workflow employed in the extraction, profiling and analysis of *N*‐ and *O*‐glycans of high‐grade serous cancers. Briefly, membrane proteins were extracted from serous cancers and released *N*‐ and *O*‐glycan alditols were separated on porous graphitized carbon (PGC) and analysed using electrospray ionization mass spectrometry (ESI‐IT‐MS/MS). Glycans were structurally characterized using tandem MS and subjected to statistical evaluations to identify discriminatory glycans implicated in the distinction of serous ovarian and peritoneal cancers. Validation of discriminatory *N*‐glycans and their corresponding glycosyltransferase genes was performed using cell lines and existing transcriptomic data set derived from a publicly available cohort.

Specimens used in this study were diagnosed by pathologists as either high‐grade serous ovarian cancer (OC) or peritoneal cancer (PC) based on their corresponding histopathological features. The membrane proteins were extracted from these tissues and the released *N*‐ and *O*‐glycan alditols were analysed using negative ion mode PGC‐LC‐ESI‐MS/MS, which resulted in the detection of well‐resolved chromatographic peaks that corresponded to specific glycan structures (Everest‐Dass *et al*., 2013a,b). Upon the identification of characteristic glycan features for each serous cancer type, glycan structural profiles were subjected to a series of statistical evaluations to potentially distinguish between serous ovarian and peritoneal cancers. For some of the tissue samples that were analysed, the diagnosis did not correspond to the location from which the tissue was derived; for example, OC specimen was obtained from an omental (omentum) metastasis and not from their corresponding primary cancer location (Table S1). Therefore, we performed two separate statistical analyses in this study: firstly on tissue samples based on diagnosis (OC *vs*. PC) and secondly, based on the site from where the cancer tissue was derived from (ovary *vs*. peritoneum *vs*. omentum). The workflow employed in this study is outlined in Fig. 1.

### 2.1 The *N*‐ and *O*‐glycan repertoires of serous adenocarcinomas

**Figure 2:**
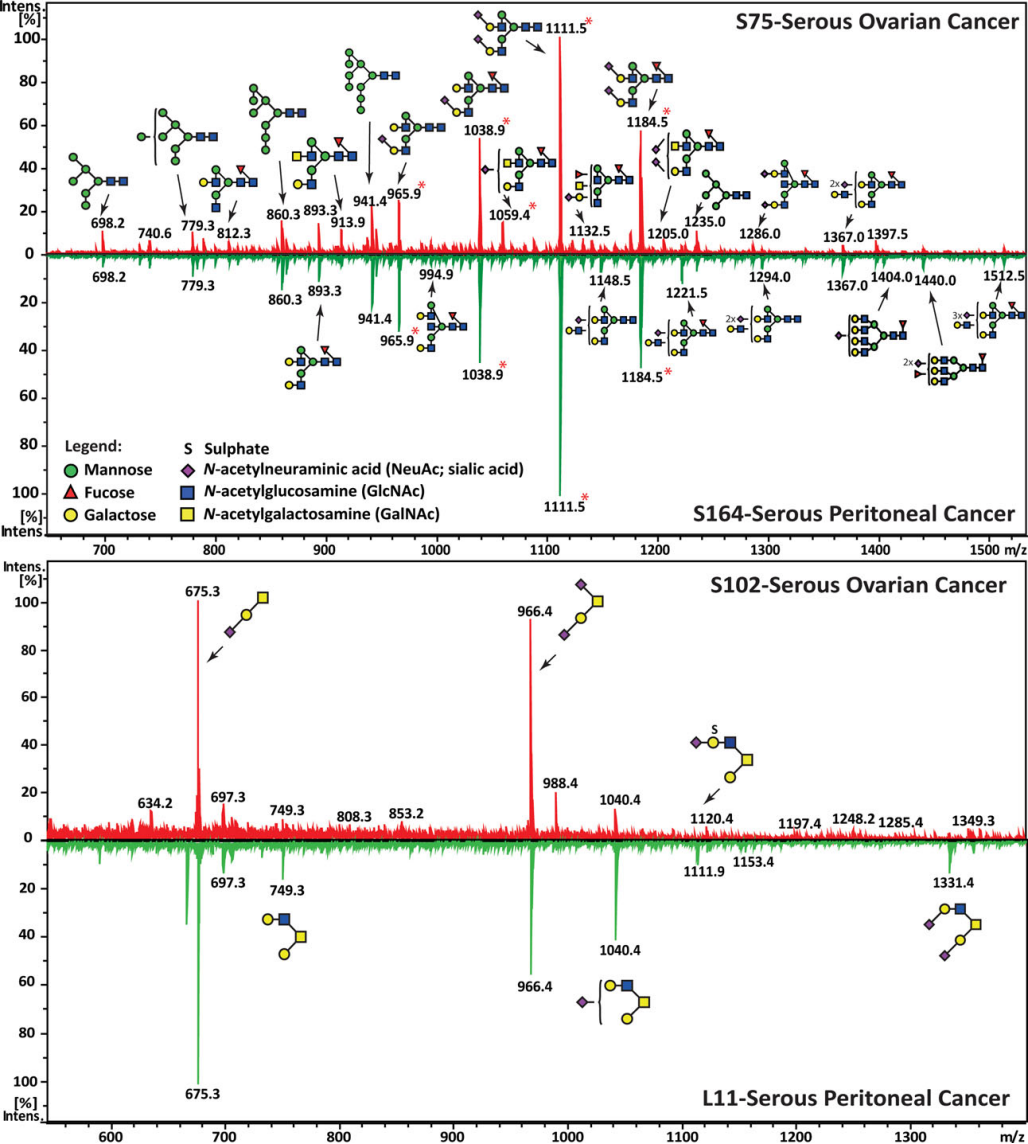
Representative average MS glycomic profiles of *N*‐ and *O*‐glycans released from membrane proteins of serous cancer tissues. (A) An overview of the representative average MS *N*‐glycan profiles in the range of *m*/*z* 640–1540 of serous ovarian (S75) and serous peritoneal (S164) cancers (LC elution time: 30–70 min). Structures were depicted according to the Essentials of Glycobiology notation (Varki *et al*., 2015) with linkage placement for sialic acid and fucose residues (where known). The *N*‐glycan structures were identified by tandem MS and are represented mainly by the doubly charged species ion, m/z [M‐2H]^2−^. Glycan masses containing isomeric structures that were resolved using retention time differences were indicated by asterisks (*). (B) Representative average MS *O*‐glycan profiles in the range of *m*/*z* 640–1340 of serous ovarian (S102) and serous peritoneal (L11) cancers (LC elution time: 30–50 min). The *O*‐glycan structures were identified by tandem MS and are represented mainly by the singly charged species ion, *m*/*z* [M‐H]^1−^.

**Figure 3:**
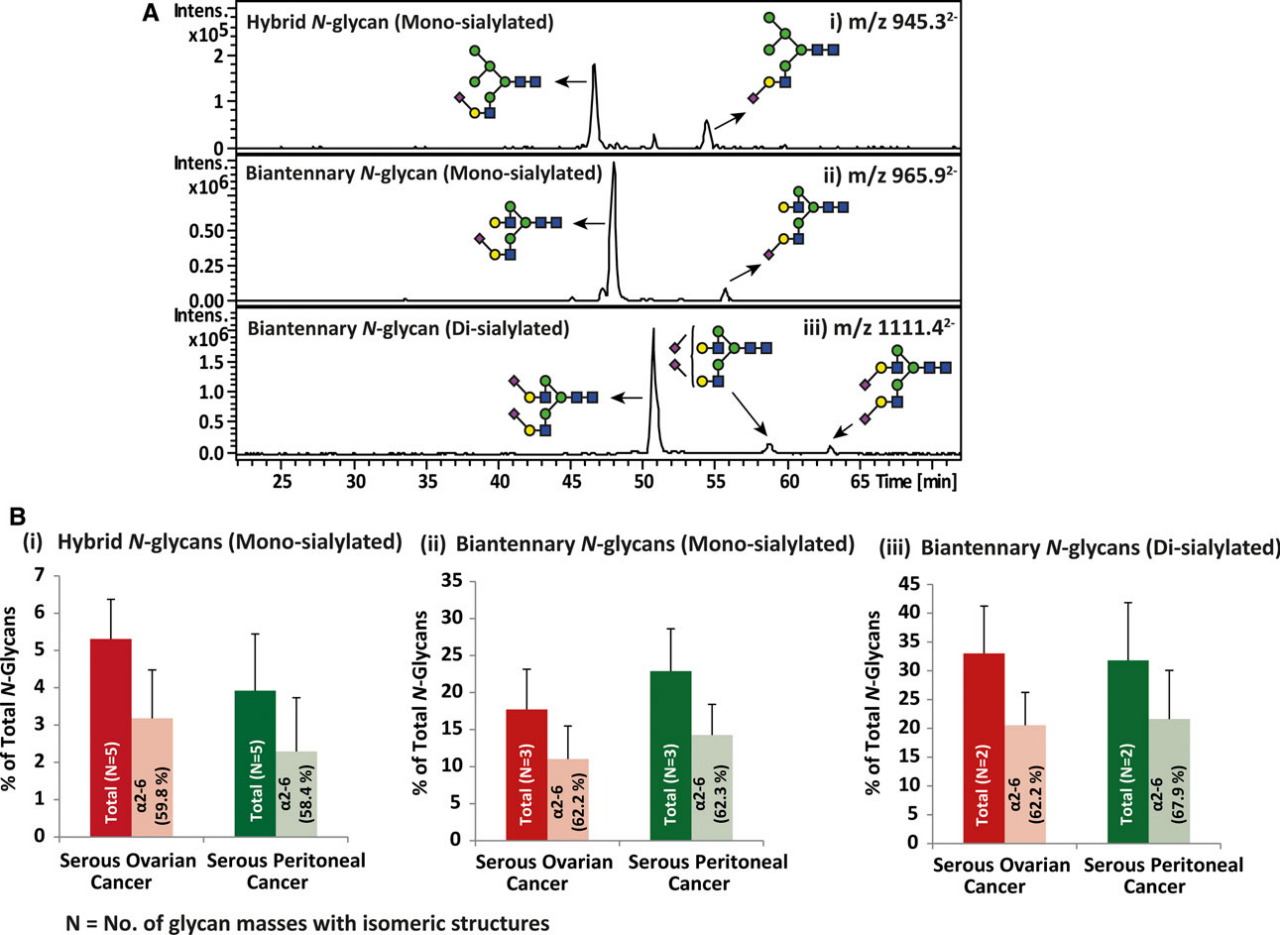
Representative extracted ion chromatograms (EIC) of mono‐ and disialylated *N*‐glycans and the corresponding relative abundance for total and α2‐6 sialylation. (A) PGC‐LC allows for the separation of α2‐6 and α2‐3 sialylated *N*‐glycans on the same underlying glycan structure based on retention time. The representative EICs depicting the separate retention times for isomers with α2‐6‐linked and α2‐3‐linked sialic acids are represented for (i) monosialylated *N*‐glycans, *m*/*z* 945.3^2−^ [(Neu5Ac)_1_(Gal)_1_(GlcNAc)_1_(Man)_2_+(Man)_3_(GlcNAc)_2_] and (ii) *m*/*z* 965.9^2−^ [(Neu5Ac)_1_(Gal)_2_ (GlcNAc)_2_ + (Man)_3_(GlcNAc)_2_] and disialylated *N*‐glycans, (iii) *m*/*z* 1111.4^2−^ [(Neu5Ac)_2_(Gal)_2_ (GlcNAc)_2_ +(Man)_3_(GlcNAc)_2_]. (B) Relative abundance for N number of *N*‐glycans bearing sialylated isomeric structures and the percentage of α2‐6 sialylation of these isomers is displayed for hybrid monosialylated (refer to Table S2: five *N*‐glycans; *m*/*z* 783.3^2−^, *m*/*z* 864.3^2−^, *m*/*z* 856.3^2−^, *m*/*z* 937.3^2−^ and *m*/*z* 945.3^2−^) (i), complex monosialylated (three *N*‐glycans; *m*/*z* 957.8^2−^, *m*/*z* 965.9^2−^ and *m*/*z* 1038.9^2−^) (ii) and complex disialylated (two *N*‐glycans; *m*/*z* 1111.4^2−^ and *m*/*z* 1184.5^2−^) (iii) for each serous cancer group. Error bars represent SD of biological replicates [serous ovarian cancer (*n* = 14) and serous peritoneal cancer (*n* = 14)]. *N = *no. of glycan masses with isomeric structures.

The glycan structures, including their isobaric isomers, were characterized by their retention time and fragment ions and were quantified based on relative integration of their extracted ion chromatographic peaks (Table S2). Their structures were classified into five major glycan types, namely high mannose, hybrid, complex neutral, complex sialylated and paucimannose (Stanley *et al*., 2009). The representative average summed MS spectra for *N*‐glycans (Fig. 2A) and *O*‐glycans (Fig. 2B) were overall similar, with the majority of the peaks in the global MS profiles of both serous cancer groups typified by sialylated *N*‐glycans, which appeared to be comparable in terms of their relative amounts. Several other peaks comprising neutral and high mannose‐type glycans were also observed, appearing at lower intensities. In total, 55 distinct *N*‐ and nine *O*‐glycan masses were identified (Table S2) across both serous cancers (OC, *n* = 14 and PC, *n* = 14) at varying relative abundance. The structures were characterized in detail based on their specific features: (a) sialylation was a major *N*‐glycan feature in both cancer types, ranging between 66.323 and 68.545% of the total *N*‐glycan structures characterized across all samples. Sialylated *N*‐glycans were further classified based on the number of sialic acid residues, in which mono‐, di‐ and trisialylated *N*‐glycans were all observed to be present (Fig. S1A,B). The four most abundant glycans comprised monosialylated (*m*/*z* 965.9^2−^ and *m*/*z* 1038.9^2−^) and disialylated (*m*/*z* 1111.5^2−^ and *m*/*z* 1184.5^2−^) *N*‐glycans in both cancers (Table S2). Likewise, for the *O*‐glycans, mono‐ and disialylation was also a prominent feature in both OC and PC cancers. Specifically, core 1 structures with *m*/*z* 675.3^1−^ [(Neu5Ac)_1_(Gal)_1_(GalNAc)_1_] and *m*/*z* 966.3^1−^ [(Neu5Ac)_2_(Gal)_1_ (GalNAc)_1_] comprised over 75% of the total ion abundance of the *O*‐glycans of OC and PC, respectively (Table S2). Several glycans with structural isomers corresponding to differences in sialic acid linkage were also observed for some of the *N*‐glycan masses. For example, the monosialylated hybrid and complex‐type *N*‐glycans with *m*/*z* 945.3^2−^ and *m*/*z* 965.9^2−^, respectively, were found as isomers with terminal α2‐3‐ or α2‐6‐linked sialic acid, which were differentiated based on their chromatographic retention times (Fig. 3Ai–ii). Likewise, the most abundant disialylated *N*‐glycan with *m*/*z* 1111.4^2−^ was found to comprise three structural isomers, in which the α2,6/α2,6‐linked isomeric structure eluted from the column earlier than the remaining two structural isomers containing α2,6/α2,3‐ and α2,3/α2,3‐linked sialic acids, respectively (Fig. 3Aiii) (Nakano *et al*., 2011). The prominence of α2‐6 sialylation on membrane proteins of serous tumours analysed in this study correlates with our previous findings reported for ovarian cancer cell lines (Anugraham *et al*., 2014). In total, 10 sialylated *N*‐glycan masses were found to contain these isomeric structures, in which five of these *N*‐glycans were monosialylated hybrid structures (*m*/*z* 783.3^2−^, *m*/*z* 856.3^2−^, *m*/*z* 864.3^2−^, *m*/*z* 937.3^2−^ and *m*/*z* 945.3^2−^), with an α2‐6 sialyl linkage, accounting for 58.465–59.864% of the total hybrid‐type isomeric structures for both serous cancers (Fig. 3Bi). Likewise, the remaining five isomeric *N*‐glycans were mono‐ (*m*/*z* 957.8^2−^, *m*/*z* 965.9^2−^ and *m*/*z* 1038.9^2−^) and disialylated (*m*/*z* 1111.4^2−^ and *m*/*z* 1184.5^2−^) complex‐type glycans, which also displayed α2‐6 sialylation for approximately 62.217–67.951% (Fig. 3Bii–iii) of the total complex‐type isomeric structures in both serous cancer groups.

(b) Biantennary and branched N‐glycans *–* Biantennary *N*‐glycans are formed through the addition of β1‐2‐linked GlcNAc (*N*‐acetylglucosamine) residues by the enzyme GlcNAcT‐II on both the α1‐3‐ and α1‐6‐linked Man residues of the *N*‐glycan core [(Man)_3_(GlcNAc)_2_], which are typically elongated with a Gal residue to form an antenna on each arm. The assignment of an antenna in this study was performed based on the presence of a complete LacNAc (Gal‐GlcNAc) disaccharide. In total, seven *N*‐glycan compositions corresponding to neutral and sialylated biantennary *N*‐glycans were observed in serous cancer tissues, accounting for approximately 53.487–58.587% of the total *N*‐glycans for both OC and PC cancers (Fig. S1C). In regard to the more highly branched *N*‐glycan structures that are synthesized by the enzymes, GlcNAcT‐IV and GlcNAcT‐V to form β1‐4‐ and β1‐6‐linked GlcNAc, respectively, eight tri‐ and three tetra‐antennary structures were identified in OC and PC cancers and comprised significantly lower relative abundance as compared to the biantennary *N*‐glycans (*P *<* *0.0001). These structures ranged between 4.402% and 5.704% of the total glycans in both serous ovarian and peritoneal cancers.

(c) Bisecting‐type biantennary N‐glycans – We also observed the presence of glycan structures corresponding to bisecting‐type biantennary *N*‐glycans. In total, there were 10 structures, of which several forms were observed such as the agalactosylated bisecting (*m*/*z* 759.9^2−^, *m*/*z* 840.8^2−^ and *m*/*z* 913.9^2−^), biantennary bisecting (*m*/*z* 921.9^2−^ and *m*/*z* 994.9^2−^), monosialylated biantennary bisecting (*m*/*z* 986.5^2−^, *m*/*z* 1059.4^2−^, *m*/*z* 1067.5^2−^ and *m*/*z* 1140.4^2−^) and disialylated biantennary bisecting (*m*/*z* 1286.0^2−^) *N*‐glycans that ranged between 6.782 and 6.894% of the total *N‐*glycans in OC and PC (Fig. S1C). A bisecting‐type *N*‐glycan is formed when a GlcNAc residue is attached to the innermost mannose of the *N*‐glycan core by the action of a specific enzyme, β1‐4‐*N*‐acetyl‐glucosaminyltransferase III (GlcNAcT‐III), encoded by the *MGAT3* gene. The presence of the ‘bisecting’ GlcNAc group is characterized by a diagnostic D‐221^−^ [203 (mass of bisecting GlcNAc) + 18 (H_2_O)] cleavage ion which occurs specifically in the negative ion MS mode. This ion can be visualized in the MS/MS fragmentation spectra of biantennary *N*‐glycans as *m*/*z* 670.2^1−^, which corresponds to the loss of the GlcNAc residue from the D ion (mass of six‐arm antennae composition and two remaining branching core Man residues), accompanied by the loss of a water molecule (Fig. S2A–C). In Fig. S2D, the D‐221^−^ ion was observed as *m*/*z* 508.3^1−^, for the agalactosylated *N*‐glycan with *m*/*z* 759.9^2−^, thereby corresponding to the mass of GlcNAc‐Man‐Man‐18‐ (H_2_O). We have previously observed bisecting‐type *N*‐glycans on ovarian cancer cell lines (SKOV3, IGROV1, A23780 and OVCAR3), but not on the normal ovarian epithelium cell lines, HOSE 6.3 and HOSE 17.1 (Anugraham *et al*., 2014), thereby validating the presence of these structures on serous cancer tissues, as evidenced in this study.

### 2.2 Elevated LacdiNAc‐type *N*‐glycans in ovarian cancer

**Figure 4:**
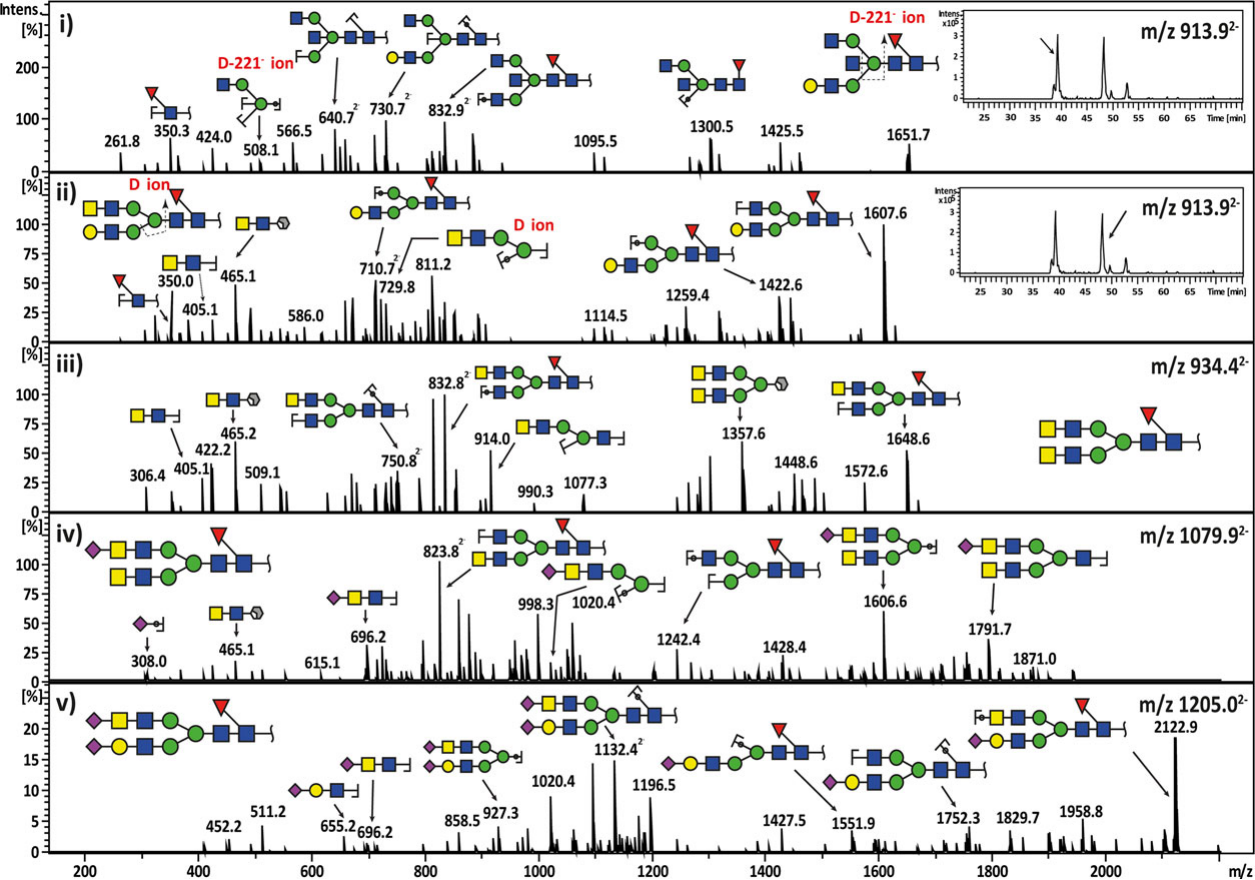
Representative MS ^2^ fragment ion spectra depicting the characterization of bisecting‐biantennary and LacdiNAc‐type *N*‐glycan structures in serous cancers. The neutral *N*‐glycan *m*/*z* 913.9^2−^ with a composition of (Hex)_1_(HexNAc)_3_(dHex)_1_ +(Man)_3_(GlcNAc)_2_ consists of two compositional isomers with separate retention times (i‐insert). The first isomer was shown to elute at 38.5 min and the tandem MS spectra corresponded to a bisecting GlcNAc‐type core‐fucosylated *N*‐glycan with a monosaccharide composition of (Gal)_1_(GlcNAc)_3_ (Fuc)_1_+(Man)_3_(GlcNAc)_2_. This was confirmed by the presence of the diagnostic D‐221^−^ cleavage ion at *m*/*z* 508.1^1−^. The second‐eluting isomer at 48 min consisted of a monosaccharide composition of (Gal)_1_ (GalNAc)_1_ (GlcNAc)_2_(Fuc)_1_+(Man)_3_(GlcNAc)_2_, which corresponded to a ‘LacdiNAc’‐type *N*‐glycan (ii‐insert). The presence of the LacdiNAc antenna (GalNAcβ1‐4GlcNAc) was indicated by the B ion at *m*/*z* 405.1^1−^ and the ^1,3^A cross‐ring cleavage ion at *m*/*z* 465.1^1−^ (‘F ion’). Several forms of LacdiNAc‐type *N*‐glycans identified in serous ovarian cancer are illustrated for neutral LacdiNAc *N*‐glycan with *m*/*z* 934.4^2−^ (iii), sialylated LacdiNAc *N*‐glycan with *m*/*z* 1079.9^2−^ (iv) and sialylated LacdiNAc/LacNAc *N*‐glycan with *m*/*z* 1205.0^2−^ (v).

Despite the overall similarity between OC and PC tissues, a comprehensive MS structural profiling of individual glycan mass signals revealed various glycan masses significantly discriminating both cancer types. In OC samples, five *N*‐glycan compositions corresponding to neutral (*m*/*z* 913.9^2−^ and *m*/*z* 934.4^2−^) and sialylated (*m*/*z* 1059.4^2−^, *m*/*z* 1079.9^2−^ and *m*/*z* 1205.0^2−^) structures were found to contain LacdiNAc (GalNAcβ1‐4GlcNAc) motifs, accounting for approximately 2.775 ± 2.005% of the total *N*‐glycans in OC, while only 0.1247 ± 1.312% was found in PC tissues (Table S2, Fig. S3). These LacdiNAc‐containing glycan masses were verified by MS/MS fragmentation patterns that differed from their compositional isomers. For instance, the *N*‐glycan mass with *m*/*z* 913.9^2−^ consists of two compositional isomers that were separated by the PGC chromatography. The early‐eluting isomer contained a bisecting‐type glycan of the same composition (Fig. 4i), while the later‐eluting isomer displayed the LacdiNAc motif (Fig. 4ii). The presence of the LacdiNAc antenna was deduced by the presence of the B ion at *m*/*z* 405.1^1−^ and the ^1,3^A cross‐ring cleavage ion at *m*/*z* 465.1^1−^. This ion, also observed in Fig. 4iii,iv, is termed as an ‘F ion’ (Harvey *et al*., 2008) and has a composition of GalNAc‐GlcNAc‐O‐CH=CH‐O‐ (GalNAc + GlcNAc + 59^1−^), which comprises the LacdiNAc disaccharide and two carbon atoms from the branching Man residue. Similarly, the *N*‐glycan with *m*/*z* 1205.0^2−^ was found to display antennae containing both the sialylated form of the LacdiNAc (Neu5Ac‐GalNAc‐GlcNAc) and the sialylated LacNAc (Neu5Ac‐Gal‐GlcNAc), observed as mass fragments of *m*/*z* 696.2^1−^ and *m*/*z* 655.2^1−^, respectively, in the MS spectra (Fig. 4v). This complex biantennary structure was present in 93% of OC samples analysed in this study. However, in the PC group, it was only found in 22% of samples.

### 2.3 The *N*‐glycome discriminates OC from PC

**Figure 5:**
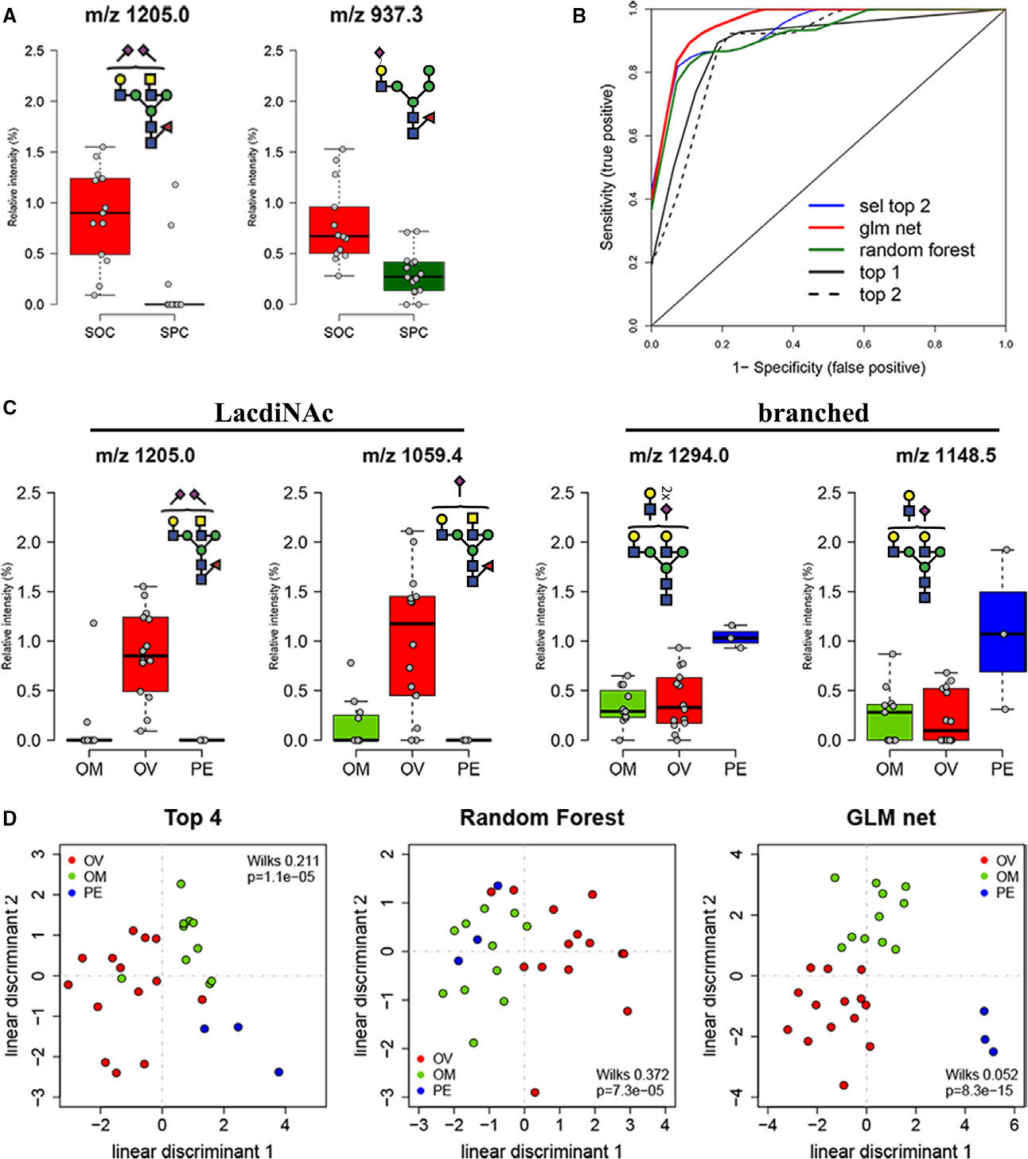
Statistical evaluation of MS‐profiled *N*‐glycan candidates involved in the discrimination of serous cancers based on diagnosis and tissue origin. (A) Box plot representing relative abundance of top two highest ranked *N*‐glycan candidates that distinguish OC and PC based on diagnosis. Data points for each serous cancer group [serous ovarian cancer (*n* = 14), serous peritoneal cancer (*n* = 14)] represent total ion intensities of glycans in each category. (B) Receiver‐operating characteristics curve for individual and feature selected glycans (refer to Table 1 for values of discriminants). (C) Box plot representing highest ranked glycan mass relative ion intensities in cancer tissues derived from omentum (OM), ovary (OV) and peritoneum (PE). Data points for each serous cancer group [ovarian (*n* = 14), omentum (*n* = 11), peritoneum (*n* = 3)] are represented. (D) Linear discriminant analysis of glycan masses selected by t statistics (Top 4, RF and GLM). Wilks lambda and corresponding *P*‐value are provided in each scatter plot.

When the relative abundance of each glycan structure was compared between OC and PC, nine *N*‐glycan structures with specific glycan structural feature epitopes were identified to be statistically significant (0.001 < *P *<* *0.05) (Table 1). Three of these *N*‐glycans (*m*/*z* 1205.0^2−^, *m*/*z* 1059.4^2−^ and *m*/*z* 1079.9^2−^) contained LacdiNAc motifs with terminal sialylation and were found to be significantly increased in OC. Several other *N*‐glycans upregulated in OC comprised of hybrid *N*‐glycans (*m*/*z* 937.3^2−^ and *m*/*z* 856.3^2−^) and a bisecting‐type sialylated biantennary *N*‐glycan (*m*/*z* 1286.0^2−^). An example of the expression levels of the glycan with sialylated LacdiNAc (*m*/*z* 1205.0^2−^) and a monosialylated hybrid glycan (*m*/*z* 937.3^2−^) in OC compared to PC tissues is shown in Fig. 5A. On the other hand, PC displayed a significant increase in bisecting‐type biantennary *N*‐glycan (*m*/*z* 994.9^2−^), agalactosylated biantennary *N*‐glycan (*m*/*z* 739.3^2−^) and monosialylated biantennary *N*‐glycan (*m*/*z* 1038.9^2−^) (Table 1). ROC curves were generated for *N*‐ and *O*‐glycan relative ion intensities that were significantly altered (*P *<* *0.05) and the resulting AUC was used as an indicator of the test accuracy. Based on the ROC analysis, two individual *N*‐glycan candidates, *m*/*z* 1205.0^2−^ (AUC 0.93) and *m*/*z* 937.3^2−^ (AUC 0.89), were able to clearly discriminate OC from PC (sensitivity: 86.7%, specificity: 92.3–100%), while the combination of both glycans revealed a higher AUC value of 0.95 and specificity (100%) (Fig. 5B, Table 1). To further evaluate the discriminatory potential of the *N‐*glycans, we performed RF and GLM classification analysis on the total *N‐*glycans released from the serous cancer tissues. In total, four structures were selected by GLM (*m*/*z* 1205.0^2−^, *m*/*z* 937.3^2−^, *m*/*z* 856.3^2−^ and *m*/*z* 994.9^2−^) and RF (*m*/*z* 937.3^2−^, *m*/*z* 856.3^2−^, *m*/*z* 739.3^2−^ and *m*/*z* 1079.9^2−^), respectively (Table 1). The corresponding AUC for the four glycans selected by GLM and RF were 0.97 (sensitivity: 93.3%, specificity: 100%) and 0.93 (sensitivity: 86.7%, specificity: 100%), respectively (Fig. 5B). These results indicate that that serous cancers of the ovary and peritoneum can be differentiated based on specific structures in their *N*‐glycan profiles, and more importantly, the selected glycan discriminants can be utilized as a panel of biomarkers to distinguish between these cancers and thus, improve their diagnosis.

**Table 1:** Statistical evaluation of N‐ and O‐glycans implicated in the distinction of serous cancers based on their diagnosis and their tissue sampling site. The table depicts the statistically significant different glycan masses and corresponding glycan structures analysed by LC‐MS/MS in tissue samples from OC and PC patients. The columns summarize statistical evaluation results (*P*‐value, selected glycan by GLM and/or RF and corresponding diagnostic performance) for two comparisons, ‘diagnosis’ (OC *vs*. PC) and ‘tissue sampling site’ (ovary *vs*. peritoneum *vs*. omentum)

Type	Glycan mass [M‐H]^−^	[M‐2H]^2−^	Structures	OC *vs*. PC (diagnosis)				AUC	Sensitivity/specificity	Ovary *vs*. peritoneum *vs*. omentum (tissue sampling site)			
				Level of significance		Feature selection				Level of significance		Feature selection	
				*P*	*P* (adj)	GLM	RF			*P*	*P* (adj)	GLM	RF
*N*‐Glycans	2411.0	1205.0	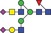	0.001	0.0041	✓		0.93	86.7/100	0.001	0.0057		
								0.95^+^	86.7/100^+^				
								0.97^GLM^	93.3/100^GLM^				
	1875.6	937.3	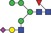	0.001	0.0107	✓	✓	0.89	86.7/92.3	0.01	0.0575	✓	✓
								0.95^+^	86.7/100^+^				
								0.97^GLM^	93.3/100^GLM^				
								0.93^RF^	86.7/100^RF^				
	2119.8	1059.4	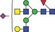	0.01	0.0777					0.001	0.0162	✓	✓
	1713.6	856.3	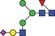	0.05	0.1086	✓	✓	0.97^GLM^	93.3/100^GLM^				
								0.93^RF^	86.7/100^RF^				
	1990.8	994.9	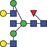	0.05	0.1808	✓		0.97^GLM^	93.3/100^GLM^	0.05	0.1555	✓	✓
	1479.6	739.3	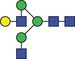	0.05	0.2147		✓	0.93^RF^	86.7/100^RF^	n.s.	n.s.		✓
	2078.8	1038.9	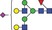	0.05	0.2159					0.05	0.1895	✓	
	2573.0	1286.0	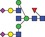	0.05	0.2850								
	2160.8	1079.9	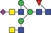	0.05	0.3737		✓	0.93^RF^	86.7/100^RF^	0.05	0.1292		
	2589.0	1294.0	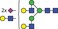							0.001	0.0162	✓	
	2298.0	1148.5	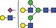							0.01	0.0283	✓	
	1828.8	913.9	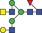							0.05	0.1243	✓	
	1891.6	945.3	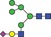							0.05	0.1880		
	2444.0	1221.5	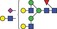							0.05	0.1292	✓	
	2881.0	1440.0	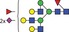							0.05	0.1785		
	3101.2	1550.1	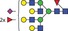							0.05	0.1219	✓	
*O*‐Glycans	749.3	–	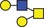							0.05	0.1219	✓	
	755.3	–	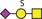							n.s.	n.s.	✓	
	1040.5	–	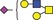							0.05	0.1880		
	1331.5	–	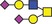							0.05	0.1292	✓	

^+^Combination AUC of top two glycan candidates; ^GLM^Combination AUC of top four glycan candidates selected by GLM; ^RF^Combination AUC of top four glycan candidates selected by RF. Legend: 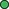, Mannose (Man); 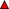, Fucose (Fuc); 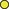, Galactose (Gal); 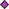, *N*‐acetylneuraminic acid (Neu5Ac); 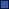, *N*‐acetylglucosamine (GlcNAc); 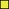, *N*‐acetylgalactosamine (GalNAc); S, Sulphate; Monosaccharide linkage: 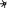.

### 2.4 LacdiNAc‐type glycans are elevated in ovarian tissue‐derived serous adenocarcinomas

The glycomic profiles of serous adenocarcinomas were also re‐evaluated based on the site from which the tumour was surgically removed, that is ovary (left and right ovary; *n* = 14), omentum (*n* = 11) and peritoneum (*n* = 3) (Table S1). We observed a high number of tissue‐based discriminatory glycans in which 13 *N*‐glycans (0.001 < *P *<* *0.05) and three *O*‐glycans (*P *<* *0.05) were identified, thereby discriminating between the three tissue origins (Table 1). Interestingly, two of the 13 *N*‐glycan candidates that contained the LacdiNAc structure (*m*/*z* 1205.0^2−^ and *m*/*z* 1059.4^2−^) were strongly associated with ovarian‐derived cancer tissues, while the remaining two candidates (*m*/*z* 1294.0^2−^ and *m*/*z* 1148.4^2−^) consisted of branched *N*‐glycans and were found to be statistically elevated in the peritoneum‐derived cancer tissues only (Fig. 5C). We further performed a linear discriminant analysis to visualize the discriminatory potential of these glycans (Fig. 5D). Interestingly, the ovarian‐derived tissues were able to be separated based on the glycan candidates selected by the RF feature selection tool, while the peritoneum‐derived tissue samples were found to be clustered separately from the omentum‐ and ovarian‐derived tissues by the GLM feature selection tool (Fig. 5D), thereby indicating that serous cancers of ovarian and peritoneum origin, regardless of their diagnosis, can also be distinguished based on their respective tissue sites.

### 2.5 *B4GALNT* gene encoding glycosyltransferases are implicated in the distinction between OC and PC

**Figure 6:**
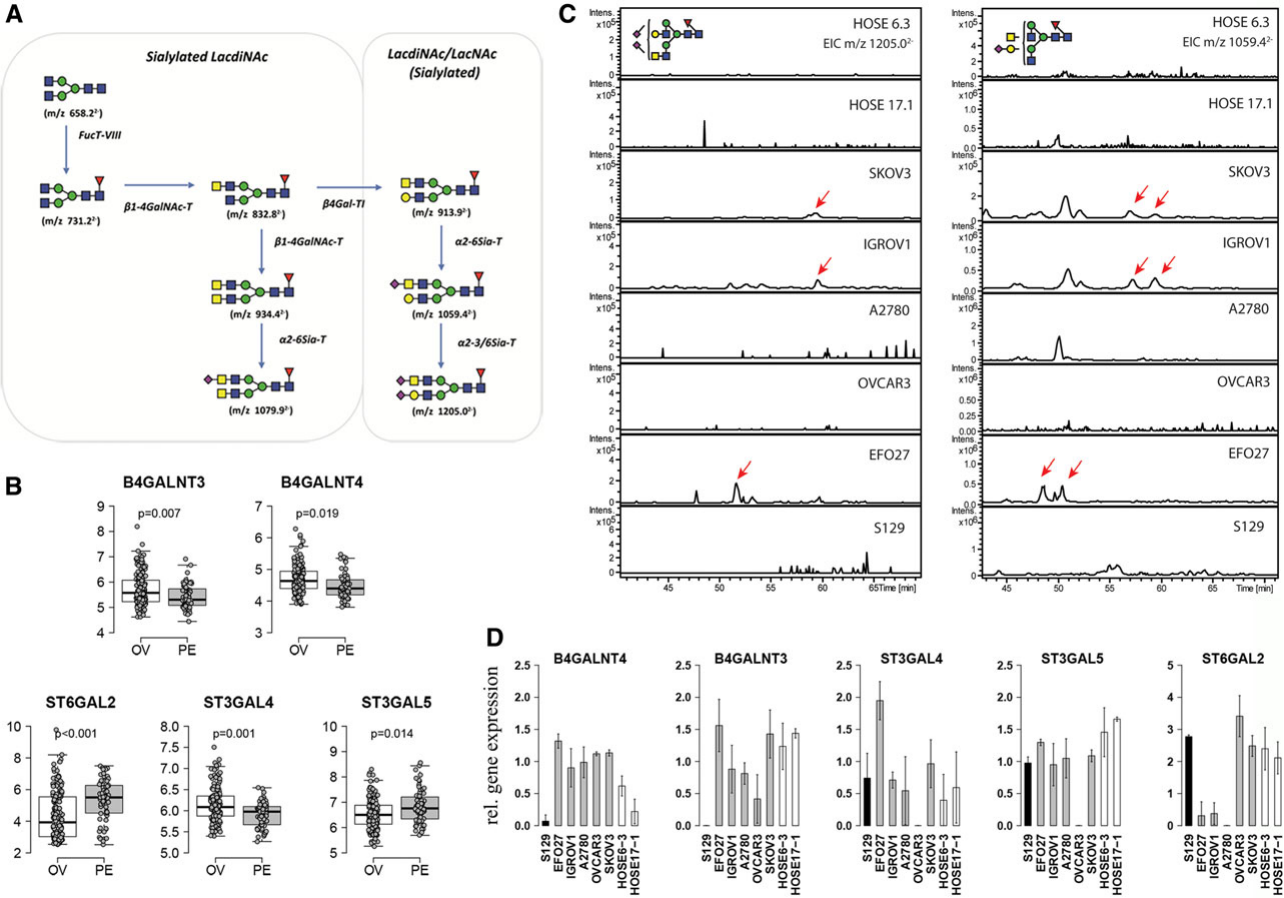
LacdiNAc is expressed in ovarian cancer cells and absent in normal surface epithelium as well as primary peritoneal cancer cells. (A) Hypothetical synthesis pathway of LacdiNAc structures on *N*‐glycoproteins. (B) Differentially expressed genes encoding glycosyltransferases involved in synthesis of LacdiNAc structures (*B4GALNT3* and *B4GALNT4*) and expression of sialyltransferase genes (*ST6GAL2*,*ST3GAL4* and *ST3GAL5*) using transcriptomic data obtained from ovarian‐derived (OV) and peritoneum‐derived (PE) cancer tissues (Tothill data set). Adjusted *P*‐values are shown in each box plot. (C) Extraction ion chromatogram to show expression of LacdiNAc structures (*m*/*z* 1205.0^2−^ and *m*/*z* 1059.4^2−^) in normal epithelial cell lines (HOSE6‐3, HOSE17‐1), ovarian cancer cells [OVCAR3, SKOV3, IGROV1, A2780 and EFO27 (shift in retention time)] and primary peritoneal cells (S129). (D) Bar graph shows relative gene expression of relevant glyco‐genes among the tested cell lines. Gene expression is displayed relative to the cell line with lowest expression.

Our glycan analysis on tissue samples revealed elevated levels of LacdiNAc‐type *N*‐glycans in both OC‐diagnosed and ovarian tissue‐derived serous adenocarcinoma samples. Glycan structures are often a consequence of altered gene expression of the corresponding glycosyltransferases responsible for their synthesis (Anugraham *et al*., 2014; Jacob *et al*., 2014b; Kawamura *et al*., 2008). We therefore accessed the transcriptomic Tothill data set to identify the expression of gene encoding glycosyltransferases potentially involved in the proposed biosynthetic pathway for the synthesis of LacdiNAc‐ and sialylated LacdiNAc‐type structures (Fig. 6A). In the Tothill data set, 232 patients fulfilled the criteria for inclusion in our analysis and consisted of 199 clinically diagnosed OC and 33 PC patients. Upon performing the initial gene expression analysis, we hypothesized that both cancers may also be differentiated based on their glyco‐gene expression profiles.

The comparison between the ovarian and peritoneal sites derived from the Tothill data set revealed five differentially expressed (*P *<* *0.05) glycosyltransferase genes (Fig. 6B). Interestingly, two of the four possible β1‐4‐acetylgalactosamine transferase genes (*B4GALNT1‐4*), *B4GALNT3* and *B4GALNT4*, were found to be significantly elevated (*P *<* *0.05) in the ovarian cancer tissues as compared to the peritoneum (Fig. 6B). We have previously reported that *N*‐glycans with LacdiNAc motif were absent in human ovarian surface epithelial cells (HOSE6‐3 and HOSE17‐1), but present in serous ovarian cancer cell lines (SKOV3 and IGROV1) (Anugraham *et al*., 2014). Hence, coupled with the new evidence from this study that the genes *B4GALNT3* and *B4GALNT4* are correlated with the presence of elevated LacdiNAc‐type *N*‐glycans on ovarian cancer tissue‐derived cell membranes, we subsequently evaluated the glycan profiles of these serous ovarian cancer cell lines, together with an additional ovarian cancer cell line (EFO27) (Fig. 6C). In addition, a primary serous peritoneal cancer cell line derived from a PC patient was also incorporated to determine whether the presence of LacdiNAc is indeed restricted to OC. To date, there is no serous peritoneal cancer cell line commercially available (Jacob *et al*., 2014c). Interestingly, both the *N*‐glycans with sialylated LacdiNAc motifs (*m*/*z* 1205.0^2−^ and *m*/*z* 1059.4^2−^) found to differentiate OC tissues were present in the serous (SKOV3 and IGROV1) and mucinous (EFO27) ovarian cancer cell lines as depicted in the extracted ion chromatograms (EIC) (Fig. 6C), while no trace of these masses was observed in the primary serous peritoneal cancer cell culture (S129) (Fig. 6C). We next established RT‐qPCR for *B4GALNT3*,* B4GALNT4*,* ST6GAL2*,* ST3GAL4*,* ST3GAL5* and three reference genes to quantify gene expression in these cell lines (Table S4). In concordance with our observations in the Tothill data set comparing both tumour sites, the peritoneal cancer cell line S129 showed lowest expression of these genes, *B4GALNT3* and *B4GALNT4*, compared to the nonovarian and ovarian cancer cell lines (*n* = 7), therefore reflecting the absence of LacdiNAc‐type *N*‐glycan structures in this primary PC cell line (S129).

As for the high expression of α2,6‐sialylated glycans observed across both cancer types, we have previously shown that *ST6GAL1*, responsible for the synthesis of α2‐6‐linked *N*‐glycans, was upregulated in all of the four cancer cell lines previously tested (Anugraham *et al*., 2014). The additional profiling of other sialyltransferase genes, *ST6GAL2, ST3GAL4* and *ST3GAL5*, was found to display varying gene expression levels between both cancer tissue types (Fig. 6B) and across all tested ovarian cancer cell lines (Fig. 6D). The differences in their expression are potentially attributed to the regulation of these enzymes across several glycan classes, including glycolipids, which were not investigated in this study.

## 3 Discussion

The field of glycomics has become increasingly investigated for its potential to unravel tumour‐specific glycans, which are associated with the cell surface of ovarian cancer cells (Drake *et al*., 2010). In the pursuit of discovering novel approaches to diagnosing the diverse group of high‐grade pelvic serous cancers, *N*‐ and *O*‐glycans were analysed from serous ovarian and peritoneal cancers using a glycomics‐based MS profiling strategy, which has been successfully employed in recent glycan biomarker studies (Alley *et al*., 2012; Biskup *et al*., 2013; Kim *et al*., 2014). Specifically, released membrane protein glycans were subjected to a rigorous, structural MS‐based characterization of glycans and classification‐based statistical tools were used to assess the diagnostic performance of these glycans, in addition to subsequent validation of specific glycosyltransferase genes implicated in their expression. To the best of our knowledge, the structural analysis of membrane protein glycosylation profiles in these cancers represents the first study of its kind, with regard to the characterization of discriminatory *N*‐ and *O*‐glycans of high‐grade serous carcinomas. While *N‐*glycosylation profiles of ovarian cancer plasma samples have been previously studied and characterized (Biskup *et al*., 2013; Mitra *et al*., 2013; Saldova *et al*., 2007, 2008), the membrane glycomic profiles of OC and PC tumour tissue samples have never been investigated and compared simultaneously. This study also showed the additional level of glycan profiling, namely via the identification of compositional and structural isomers, which is required for the determination of low abundance discriminatory structures in serous cancers of differing cellular origins.

The specific cellular glycosylation and phenotypic alterations that occur as a result of molecular genetic mutations are unknown, particularly for these cancers. As opposed to ovarian cancers which have been extensively studied and characterized by high levels of genetic instability and frequent mutations of *TP53* (Jarboe *et al*., 2008; Kurman and Shih Ie, 2011), primary serous peritoneal cancers are less frequently investigated (Jordan *et al*., 2008; Nik *et al*., 2014) and are thought to share a common aetiology with ovarian cancers based on their close histological and clinical similarities (Kessler *et al*., 2013). It is therefore not surprising that this close association between these serous cancers was also reflected in their membrane protein *N*‐ and *O*‐glycomic profiles, as evidenced broadly by the similar abundance when grouped into their glycan types. It is also interesting to note that the membrane proteins of the tumour tissues displayed abundant sialylation, a feature that has also been observed in a recent study using high‐resolution MALDI‐MS imaging (Everest‐Dass *et al*., 2016). In this study, the authors noted, however, that the mono‐ and disialylated glycans were predominantly located in the stromal region of ovarian tumour tissue cross‐sections. Despite their similarities in overall glycan profiles, we demonstrate that the glycosylation of cell membrane proteins of cancer cells is specific to the disease and the location where the tumour sample was derived from. In particular, structurally related *N*‐glycans containing LacdiNAc‐type motifs, which have not been previously reported in membrane proteins of serous cancers, were found to be highly specific for OC. This nonreducing end terminal group is rare on mammalian glycoproteins, and can be further modified with sialic acid, fucose and sulfate (Hirano *et al*., 2014). In agreement with this finding, we have previously identified LacdiNAc‐type *N*‐glycans in ovarian cancer cell lines but not on normal HOSE cell line membrane proteins (Anugraham *et al*., 2014) and this glycan motif was also reported to be a tumour‐specific glycan in breast and ovarian cancer cell lines investigated in a separate study (Guo and Abbott, 2015). In this tissue‐based study, we identified three sialylated LacdiNAc‐type *N*‐glycans (*m*/*z* 1205.0^2−^, *m*/*z* 1059.4^2−^ and *m*/*z* 1079.9^2−^) that were capable of distinguishing OC from PC, and these glycans, together with an additional nonsialylated LacdiNAc‐type *N*‐glycan (*m*/*z* 913.9^2−^), were all found to be associated with ovarian cancer‐derived tissues only. These findings point to the increasing evidence that glycans are indeed tissue specific and potentially offer a novel, yet exciting avenue for targeted therapies/diagnostics.

Most mammalian *N*‐glycans contain LacNAc (Galβ1‐3/4GlcNAc) antennae, which form biantennary, or branched tri‐ and tetra‐antennary structures that are usually capped by sialylation or extended to form poly‐LacNAc chains. The less common LacdiNAc‐type glycan, however, is synthesized by the action of specific β4‐GalNAc transferases (e.g. B4GALNT3 or B4GALNT4) (Gotoh *et al*., 2004; Sato *et al*., 2003). Interestingly, while no trace of LacdiNAc *N*‐glycans was detected in the peritoneal cancer cell line, the nonserous, mucinous‐type ovarian cancer cell line EFO27 was also found to display this motif, thereby indicating that LacdiNAc‐type *N*‐glycans could be largely associated with ovarian‐derived cancer tissue. Nevertheless, further analyses on nonserous‐type cell lines need to be performed to determine whether this epitope is nonserous specific. It is also interesting to note that these unique structures have never been reported in the sera of patients with ovarian cancer (Alley *et al*., 2012; Hua *et al*., 2013; Saldova *et al*., 2007), possibly due to the low abundance of these *N*‐glycan epitopes. In regard to the glycosyltransferase expression extrapolated from the Tothill data set, there seems to be an increased expression of *B4GALNT3* and *B4GALNT4* in serous tumours derived from the ovary as compared to the peritoneum, consistent with our findings in this study. It is evident that a closer investigation into the specific regulation of the LacdiNAc‐type pathway in high‐grade serous cancers (HGSCs), particularly in cancers originating from the ovary, is warranted.

The peritoneal origin of serous cancers remains unclear as the normal peritoneum is composed of a layer of mesothelium that forms the lining of the abdominal cavity and can undergo malignant transformation to form a tumour entirely different from serous peritoneal carcinoma (Seidman *et al*., 2011). In fact, the serous‐type epithelium from which serous peritoneal cancer is thought to be derived, positioned just underneath the peritoneal surface, is ciliated and considered identical to that of the normal fallopian tube. In regard to their glycan profiles, several glycosylation features were shown to be upregulated in PC and implicated in the distinction between ovary‐, omentum‐ and peritoneum‐derived tissues. Most notably, in peritoneal/omentum‐derived serous cancer tissues, structures such as the bisecting GlcNAc‐type (*m*/*z* 994.9^2−^), branched *N*‐glycans with increased Gal‐GlcNAc units (*m*/*z* 1294.0^2−^ and *m*/*z* 1148.4^2−^) and sialylated core 2 *O*‐glycan (*m*/*z* 1331.5^2−^) were all found to be discriminatory. Bisecting GlcNAc *N*‐glycan structures have been recently associated with survival in ovarian cancer and were found to result from an epigenetically regulated *MGAT3* expression (Kohler *et al*., 2016). Our findings may indicate that bisecting GlcNAc plays a role in ovarian cancer metastasis, possibly to various sites of the omentum/peritoneum. The appearance of these glycan structures with greater complexity, that is branching patterns in the peritoneal/omentum‐derived tissues, might be an adaptation of cancer cells at the metastatic site to maintain tumour growth, which has been demonstrated in the case of brain metastasis for patients with breast cancer (Bos *et al*., 2009).

In conclusion, our study provides the first molecular evidence that OC and PC represent two distinct diseases, reflected by differentially expressed glycans attached to glycoproteins and their corresponding altered glycosyltransferase gene levels. While at present there is increasing evidence that HGSCs originate from the fimbrial end of the tube, this is the first study performed to clearly demonstrate that OC and PC need to be regarded as distinct entities. It is possible that the two cancer types may have been derived originally from the fallopian tube and have metastasized and adapted different tissue glycosylation pathways, thereby displaying glycan features that are representative of their respective metastatic sites. Our preliminary analyses of omentum/peritoneal samples from an ovarian cancer case and ovarian‐derived sample from peritoneal cancer case indicate that the glycan profiles exhibit similarity based on their site of origin, independent of the initial diagnosis. Ideally, knowledge of the glycomics profiles of cancer tissue samples derived from different locations including the metastasized sites will also provide mechanistic insights in future. Alternatively, it is possible that a specific subpopulation of serous tubal intraepithelial cancer cells, with a more mesenchymal‐like differentiation, may trigger the omental or peritoneal invasion rather than the ovarian. The detection of these underlying morphological changes is challenging and may further complicate the clinical diagnosis of advanced‐stage serous cancers, as well as the subsequent treatment and management of these aggressive cancers. It is therefore important that future clinical considerations incorporate the origin of each cancer type, and it is envisaged that the understanding of the differences in specific membrane glycosylation determinants between these serous cancers could provide alternative approaches for the disease to be diagnosed and ultimately facilitate the development of tailored ovarian cancer therapeutics.

## 4 Materials and methods

### 4.1 Biospecimens collection

Patients diagnosed with HGSCs were either admitted with an adnexal mass to the Department of Gynecology, University Hospital Zurich, Switzerland; Limmattalspital, Schlieren, Switzerland; the Gynecological Cancer Centre of the Royal Hospital for Women, Randwick, Australia; or the Gynecological Cancer Centre of the John Hunter Hospital in Newcastle, Australia. All patients were prospectively included after giving informed consent in accordance with ethical regulations (SPUK, Canton of Zurich, Switzerland, Hunter Area Research Ethics 04/04/07/3.04; South Eastern Sydney Illawarra HREC/AURED Ref: 08/09/17/3.02). Tissue and blood samples were collected during surgery. All samples were selected from chemonaïve tumours. Tissue was kept in RNA later solution (Life Technologies, Zug, Switzerland) and stored at −80 °C until further use. The processing of blood plasma samples was performed constantly on ice within three hours after collection as previously described (Jacob *et al*., 2011, 2012, 2014b). All clinicopathological data were incorporated in a specifically designed in‐house database (‘PEROV’; Microsoft Access, Microsoft, USA). The diagnosis of these cancers as either serous ovarian (OC) or serous peritoneal (PC) cancers and their corresponding histopathological features were independently re‐evaluated (Table S1).

### 4.2 Cancer tissue processing

Tissue processing was carried out using standardized clinical and ethical protocols approved through independent, board‐approved institutions during the entire collection period (Macquarie University‐Ref: 26/09/11/5201100778). Intact tissue samples (~ 40 mg) were removed from RNAlater^®^ solution (Ambion, Thermo Fisher Scientific, Waltham, MA, USA) using sterile forceps, transferred to a Ultracell MC 0.45‐μ filter unit placed in a 1.5‐mL Eppendorf collection tube as previously described (Rader *et al*., 2008). An aliquot of 500 μL of ice‐cold acetonitrile/water (80 : 20) was added to the tissue samples and resulting RNAlater^®^ ice crystals were precipitated by centrifugation. The biphasic liquid in the collection tube was discarded and several washings with acetonitrile/water were repeated until there was no visible biphasic layer or ice crystals observed.

### 4.3 Membrane protein extraction and glycan release from membrane proteins

Tissues were washed in PBS prior to the Triton X‐114‐based membrane protein extraction protocol carried out as previously described (Lee *et al*., 2009). Briefly, cut tissues were homogenized in lysis buffer and the lysed cells in the supernatant were further sedimented by ultracentrifugation. The membrane cell pellet was resuspended in Tris‐binding buffer prior to the addition of Triton X‐114 for phase partitioning of membrane proteins. The membrane proteins in the lower detergent layer were precipitated with acetone, pelleted by centrifugation and solubilized in 8 m urea. *N*‐ and *O*‐Glycans were prepared as previously described (Jensen *et al*., 2012). Briefly, membrane proteins and glycoprotein standard (10 μg of fetuin) were spotted onto a polyvinylidene difluoride (PVDF) membrane, dried overnight at room temperature and stained. Protein spots were cut, placed in separate wells of a 96‐well microtiter plate and treated with PNGase F enzyme. The released *N*‐glycans were collected and treated with ammonium acetate (pH 5.0) to regenerate the reducing terminus and later reduced to alditols with sodium borohydride. For *O*‐glycans, the remaining PVDF spots were treated with sodium borohydride and released via reductive β‐elimination. *N*‐ and *O*‐Glycan alditols were desalted using cation‐exchange resin beads (AG50W‐X8) and residual borate was removed by adding methanol. The purified *N*‐ and *O*‐glycan alditols were resuspended in milliQ water prior to mass spectrometry analysis.

### 4.4 Mass spectrometric analysis of *N*‐ and *O*‐glycans

The separation of glycans was performed on a Hypercarb porous graphitized carbon capillary column (5 μm Hypercarb KAPPA, 180 μm × 100 mm; Thermo Hypersil, Runcorn, UK) over a linear gradient for 85 min of 0–45% and 45 min of 0–90% (v/v) acetonitrile/10 mm ammonium bicarbonate for *N*‐ and *O*‐glycans, respectively. The MS instrument used in this study consisted of an ion trap mass spectrometer (LC/MSD Trap XCT Plus Series 1100; Agilent Technologies, Santa Clara, CA, USA) connected to an ESI source (Agilent 6330) and an Agilent autosampler (Agilent 1100). The temperature of the transfer capillary was maintained at 300 °C and the capillary voltage was set at 3 kV with helium as the collision gas. The sample injection volume was 7 and 4 μL for *N*‐ and *O*‐glycans, respectively, and the flow rate was set at 2 μL·min^−1^. *N*‐ and *O*‐Glycans were detected in negative ionization mode as [M‐H]^1−^ and [M‐2H]^2−^ ions within the mass range of *m*/*z* 200–2200. MS data were integrated using compass data analysis version 4.0 software (Bruker Daltonics, Billerica, MA, USA). Monoisotopic masses detected in negative mode were assigned to possible monosaccharide compositions using the GlycoMod tool (Cooper *et al*., 2001) available on the ExPASy server (http://au.expasy.org/tools/glycomod) with a mass tolerance of ± 0.5 Da. Glycan structures were identified based on manual annotation of tandem MS fragmentation spectra and further characterized with the aid of software‐generated mass fragments using GlycoWorkBench (Ceroni *et al*., 2008). Verification of the proposed structures was also carried out using UniCarb DB (Campbell *et al*., 2014b; Hayes *et al*., 2011), a web‐based LC‐MS/MS database containing fragmentation and retention times of previously reported *N*‐ and *O*‐glycans (Campbell *et al*., 2014a).

### 4.5 Cell culture

Ovarian cancer cell lines (IGROV1, A2780, SKOV3, EFO27 and OVCAR3) and human ovarian surface epithelial cells (HOSE6‐3 and HOSE17‐1) were grown in RPMI‐1640 media (Sigma‐Aldrich, Buchs, Switzerland) supplemented with 10% (v/v) fetal bovine serum (FBS; Sigma‐Aldrich), penicillin (100 U·mL^−1^) and streptomycin (100 μg·mL^−1^) (Sigma‐Aldrich). They were incubated at 37 °C in a 95% humidified atmosphere containing 5% CO_2_. Cells were propagated at about 80% confluence every two to three days and tested routinely for mycoplasma infection (Uphoff and Drexler, 2005). Cultured cells were harvested using Cell Dissociation Solution nonenzymatic 1× (Sigma). In regard to culture of primary serous peritoneal cancer cells, ascites was collected at the time of surgery, centrifuged at 3000 ***g*** for 10 min and cultured in DMEM/F12 (FBS, Sigma‐Aldrich) supplemented with 20% (v/v) fetal bovine serum, penicillin (100 U·mL^−1^) and streptomycin (100 μg·mL^−1^).

### 4.6 Reverse transcription quantitative polymerase chain reaction (RT‐qPCR)

In order to examine the expression of ‘glyco’ and reference genes, cells were grown in six‐well plates (NUNC, Thermo Fisher Scientific, Roskilde, Denmark). Cells were washed twice using DPBS (Gibco, Sigma‐Aldrich, Basel, Switzerland) and lysed, and total RNA was isolated using NucleoSpin RNAII kit (MACHEREY‐NAGEL, Macherey‐Nagel AG, Oensingen, Switzerland). RNA was eluted in 50 μL RNase‐free water. After extraction and concentration, total RNA was measured at *A*_260/230 nm_ and *A*_260/280 nm_ using NanoDrop ND‐1000 spectrophotometer (Thermo Fisher Scientific). The RNA integrity of samples was confirmed by electropherograms (Agilent 2100 Bioanalyzer, Agilent Technologies (Schweiz) AG Life Sciences & Chemical Analysis, Basel, Switzerland). An amount of 1 μg of total RNA was reverse‐transcribed using the iScript Reverse Transcription Supermix for RT‐qPCR (Bio‐Rad Laboratories AG, Cressier, Switzerland) and complementary DNA (cDNA) was stored at −20 °C until use. RT‐qPCR was performed in concordance to MIQE guidelines (Bustin *et al*., 2009; Jacob *et al*., 2014b). qPCR was performed on ‘glyco’ genes (*B4GALNT3*,* B4GALNT4*,* ST3GAL4*,* ST3GAL5* and *ST6GAL2*) and three putative reference genes (*SDHA*,* YWHAZ* and *HSPCB*) (Tables S3 and S4). Primers specific to ‘glyco’ genes were designed using QuantPrime (Arvidsson *et al*., 2008). qPCR was performed on Applied Biosystems ViiA^™^ Real‐Time PCR System (Life Technologies) in 384‐well microtitre plates (Life Technologies). To achieve reliable gene expression patterns, all ‘glyco’ gene expressions were normalized against the logarithmic mean of the three reference genes.

### 4.7 Data acquisition and bioinformatical analysis

For the exploratory genetic analyses, we used a large publicly available Affymetrix microarray data set involving patients with OC and PC that provided associated clinical information. As most data sets did not pass the selection criteria of including both OC and PC, the Tothill analysis was selected as it was the only one that fulfilled these criteria (Tothill *et al*., 2008). CEL files (Affymetrix U133, *n* = 285) and clinical data were downloaded from the Gene Expression Omnibus (GSE9899). The tumour location for sampling was provided in the supplementary data of the original publication (Tothill *et al*., 2008) and this information was incorporated into our study. We included only high‐grade serous ovarian or peritoneal cancers of advanced FIGO stages into our analysis to provide a homogeneous group. Statistical analysis and figures were obtained through the use of the software r version 3.1.3 (www.R-project.org). Following R packages were therefore applied: ‘cgdsr’, ‘catspec’, ‘compareGroups’, ‘party’, ‘ggplot2’ and ‘Rcpp’. The ‘limma’ package from bioconductor open source software for bioinformatics (r statistical software) was used to identify differentially expressed genes between study groups. *P*‐values were adjusted by the Benjamini–Hochberg false discovery rate method. In order to perform stable feature selections for classification and prediction of subgroups, two popular methods were selected, random forest (RF) and penalized generalized linear model (GLM). RF was performed using the package ‘randomForestSRC’. Penalized multinomial regression (Glmnet) was performed using the package ‘glmnet’ within the r software. The comparison of two algorithm guarantees a certain stability of the selected features. Subsequent ROC curves with corresponding AUC were estimated using the R package ‘pROC’. Additional linear discriminant analysis (LDA) and multivariate ANOVA were performed to test and visualize the performance of the feature selections (R package ‘lda’, ‘manova’). Survival analysis was conducted using Kaplan–Meier curves with corresponding log‐rank tests (R package ‘Survival’). A *P*‐value < 0.05 was considered significant but interpreted exploratory. All evaluations were made using r version 3.1.3 and bioconductor.

## Author contributions

VHS, FJ, MA and NHP designed research; MA and FJ performed experiments; VHS, NHP, NVB, DF, AF, NFH and MMH contributed and prepared samples, reagents and analytic tools; FJ, MA, AVED and AS analysed data; MA, FJ, NHP, NFH and VHS wrote the manuscript.

## Supporting information


**Fig. S1.** Relative quantitation of abundances of sialylation levels (mono‐, di‐ and tri‐) and branching patterns of *N*‐ and *O*‐glycans.
**Fig. S2.** Representative MS^2^ fragment ion spectra depicting the diagnostic ions of bisecting GlcNAc type *N*‐glycans in serous cancers.
**Fig. S3.** Representative extracted ion chromatograms (EIC) of glycan masses bearing terminal LacdiNAc (GalNAc‐GlcNAc) at *m*/*z* 913.9^2−^ and sialylated LacdiNAc (Neu5Ac‐GalNAc‐GlcNAc) at *m*/*z* 1205.0^2−^ serous ovarian and peritoneal cancers.
**Table S1.** Source and clinico‐pathological information of serous cancer specimens.
**Table S2.** Proposed *N*‐ and *O*‐glycan structures detected on the membrane proteins of serous cancers derived from the ovary and peritoneum.
**Table S3.** QPCR parameters providing the standard curve parameters for each primer pair on 8 targeted genes.
**Table S4.** QPCR parameters providing the standard curve parameters for each primer pair on 8 targeted genes.

## References

[mol212134-bib-0001] Abbott KL , Lim JM , Wells L , Benigno BB , McDonald JF and Pierce M (2010) Identification of candidate biomarkers with cancer‐specific glycosylation in the tissue and serum of endometrioid ovarian cancer patients by glycoproteomic analysis. Proteomics 10, 470–481. 19953551 10.1002/pmic.200900537 PMC4932840

[mol212134-bib-0002] Abbott KL , Nairn AV , Hall EM , Horton MB , McDonald JF , Moremen KW , Dinulescu DM and Pierce M (2008) Focused glycomic analysis of the N‐linked glycan biosynthetic pathway in ovarian cancer. Proteomics 8, 3210–3220. 18690643 10.1002/pmic.200800157 PMC3970323

[mol212134-bib-0003] Alley WR Jr , Vasseur JA , Goetz JA , Svoboda M , Mann BF , Matei DE , Menning N , Hussein A , Mechref Y and Novotny MV (2012) N‐linked glycan structures and their expressions change in the blood sera of ovarian cancer patients. J Proteome Res 11, 2282–2300. 22304416 10.1021/pr201070k PMC3321095

[mol212134-bib-0004] Anugraham M , Jacob F , Nixdorf S , Everest‐Dass AV , Heinzelmann‐Schwarz V and Packer NH (2014) Specific glycosylation of membrane proteins in epithelial ovarian cancer cell lines: glycan structures reflect gene expression and DNA methylation status. Mol Cell Proteomics 13, 2213–2232. 24855066 10.1074/mcp.M113.037085 PMC4159645

[mol212134-bib-0005] Arvidsson S , Kwasniewski M , Riano‐Pachon DM and Mueller‐Roeber B (2008) QuantPrime – a flexible tool for reliable high‐throughput primer design for quantitative PCR. BMC Bioinformatics 9, 465. 18976492 10.1186/1471-2105-9-465 PMC2612009

[mol212134-bib-0006] Bandera CA , Muto MG , Schorge JO , Berkowitz RS , Rubin SC and Mok SC (1998a) BRCA1 gene mutations in women with papillary serous carcinoma of the peritoneum. Obstet Gynecol 92, 596–600. 9764635 10.1016/s0029-7844(98)00223-3

[mol212134-bib-0007] Bandera CA , Muto MG , Welch WR , Berkowitz RS and Mok SC (1998b) Genetic imbalance on chromosome 17 in papillary serous carcinoma of the peritoneum. Oncogene 16, 3455–3459. 9692553 10.1038/sj.onc.1201901

[mol212134-bib-0008] Barda G , Menczer J , Chetrit A , Lubin F , Beck D , Piura B , Glezerman M , Modan B and Sadetzki S (2004) Comparison between primary peritoneal and epithelial ovarian carcinoma: a population‐based study. Am J Obstet Gynecol 190, 1039–1045. 15118638 10.1016/j.ajog.2003.09.073

[mol212134-bib-0009] Biskup K , Braicu EI , Sehouli J , Fotopoulou C , Tauber R , Berger M and Blanchard V (2013) Serum glycome profiling: a biomarker for diagnosis of ovarian cancer. J Proteome Res 12, 4056–4063. 23889230 10.1021/pr400405x

[mol212134-bib-0010] Bos PD , Zhang XH , Nadal C , Shu W , Gomis RR , Nguyen DX , Minn AJ , van de Vijver MJ , Gerald WL , Foekens JA *et al* (2009) Genes that mediate breast cancer metastasis to the brain. Nature 459, 1005–1009. 19421193 10.1038/nature08021 PMC2698953

[mol212134-bib-0011] Bustin SA , Benes V , Garson JA , Hellemans J , Huggett J , Kubista M , Mueller R , Nolan T , Pfaffl MW , Shipley GL *et al* (2009) The MIQE guidelines: minimum information for publication of quantitative real‐time PCR experiments. Clin Chem 55, 611–622. 19246619 10.1373/clinchem.2008.112797

[mol212134-bib-0012] Campbell MP , Nguyen‐Khuong T , Hayes CA , Flowers SA , Alagesan K , Kolarich D , Packer NH and Karlsson NG (2014a) Validation of the curation pipeline of UniCarb‐DB: building a global glycan reference MS/MS repository. Biochem Biophys Acta 1844, 108–116. 23624262 10.1016/j.bbapap.2013.04.018

[mol212134-bib-0013] Campbell MP , Peterson R , Mariethoz J , Gasteiger E , Akune Y , Aoki‐Kinoshita KF , Lisacek F and Packer NH (2014b) UniCarbKB: building a knowledge platform for glycoproteomics. Nucleic Acids Res 42, D215–D221. 24234447 10.1093/nar/gkt1128 PMC3964942

[mol212134-bib-0014] Ceroni A , Maass K , Geyer H , Geyer R , Dell A and Haslam SM (2008) GlycoWorkbench: a tool for the computer‐assisted annotation of mass spectra of glycans. J Proteome Res 7, 1650–1659. 18311910 10.1021/pr7008252

[mol212134-bib-0015] Chandrasekaran EV , Xue J , Neelamegham S and Matta KL (2006) The pattern of glycosyl‐ and sulfotransferase activities in cancer cell lines: a predictor of individual cancer‐associated distinct carbohydrate structures for the structural identification of signature glycans. Carbohydr Res 341, 983–994. 16545347 10.1016/j.carres.2006.02.017

[mol212134-bib-0016] Chen LM , Yamada SD , Fu YS , Baldwin RL and Karlan BY (2003) Molecular similarities between primary peritoneal and primary ovarian carcinomas. Int J Gynecol Cancer 13, 749–755. 14675310 10.1111/j.1525-1438.2003.13605.x

[mol212134-bib-0017] Christiansen MN , Chik J , Lee L , Anugraham M , Abrahams JL and Packer NH (2014) Cell surface protein glycosylation in cancer. Proteomics 14, 525–546. 24339177 10.1002/pmic.201300387

[mol212134-bib-0018] Coleman MP , Forman D , Bryant H , Butler J , Rachet B , Maringe C , Nur U , Tracey E , Coory M , Hatcher J *et al* (2011) Cancer survival in Australia, Canada, Denmark, Norway, Sweden, and the UK, 1995‐2007 (the International Cancer Benchmarking Partnership): an analysis of population‐based cancer registry data. Lancet 377, 127–138. 21183212 10.1016/S0140-6736(10)62231-3 PMC3018568

[mol212134-bib-0019] Cooper CA , Gasteiger E and Packer NH (2001) GlycoMod – a software tool for determining glycosylation compositions from mass spectrometric data. Proteomics 1, 340–349. 11680880 10.1002/1615-9861(200102)1:2<340::AID-PROT340>3.0.CO;2-B

[mol212134-bib-0020] Cummings RD and Pierce JM (2014) The challenge and promise of glycomics. Chem Biol 21, 1–15. 24439204 10.1016/j.chembiol.2013.12.010 PMC3955176

[mol212134-bib-0021] Drake PM , Cho W , Li B , Prakobphol A , Johansen E , Anderson NL , Regnier FE , Gibson BW and Fisher SJ (2010) Sweetening the pot: adding glycosylation to the biomarker discovery equation. Clin Chem 56, 223–236. 19959616 10.1373/clinchem.2009.136333 PMC2849286

[mol212134-bib-0022] Everest‐Dass AV , Abrahams JL , Kolarich D , Packer NH and Campbell MP (2013a) Structural feature ions for distinguishing N‐ and O‐linked glycan isomers by LC‐ESI‐IT MS/MS. J Am Soc Mass Spectrom 24, 895–906. 23605685 10.1007/s13361-013-0610-4

[mol212134-bib-0023] Everest‐Dass AV , Briggs MT , Kaur G , Oehler MK , Hoffmann P and Packer NH (2016) N‐glycan MALDI imaging mass spectrometry on formalin‐fixed paraffin‐embedded tissue enables the delineation of ovarian cancer tissues. Mol Cell Proteomics 15, 3003–3016. 27412689 10.1074/mcp.M116.059816 PMC5013313

[mol212134-bib-0024] Everest‐Dass AV , Kolarich D , Campbell MP and Packer NH (2013b) Tandem mass spectra of glycan substructures enable the multistage mass spectrometric identification of determinants on oligosaccharides. Rapid Commun Mass Spectrom 27, 931–939. 23592194 10.1002/rcm.6527

[mol212134-bib-0025] Fishman DA and Bozorgi K (2002) The scientific basis of early detection of epithelial ovarian cancer: the National Ovarian Cancer Early Detection Program (NOCEDP). Cancer Treat Res 107, 3–28. 11775458 10.1007/978-1-4757-3587-1_1

[mol212134-bib-0026] Gao B , Lindemann K , Anderson L , Fereday S , Hung J , Alsop K , Tothill RW , Gebski V , Kennedy C , Balleine RL *et al*(2016) Serous ovarian and primary peritoneal cancers: a comparative analysis of clinico‐pathological features, molecular subtypes and treatment outcome. Gynecol Oncol 142, 458–464. 27444035 10.1016/j.ygyno.2016.06.023

[mol212134-bib-0027] Gotoh M , Sato T , Kiyohara K , Kameyama A , Kikuchi N , Kwon YD , Ishizuka Y , Iwai T , Nakanishi H and Narimatsu H (2004) Molecular cloning and characterization of beta1,4‐N‐acetylgalactosaminyltransferases IV synthesizing N, N’‐diacetyllactosediamine. FEBS Lett 562, 134–140. 15044014 10.1016/S0014-5793(04)00219-4

[mol212134-bib-0028] Guo H and Abbott KL (2015) Functional impact of tumor‐specific N‐linked glycan changes in breast and ovarian cancers. Adv Cancer Res 126, 281–303. 25727151 10.1016/bs.acr.2014.11.006

[mol212134-bib-0029] Harvey DJ , Royle L , Radcliffe CM , Rudd PM and Dwek RA (2008) Structural and quantitative analysis of N‐linked glycans by matrix‐assisted laser desorption ionization and negative ion nanospray mass spectrometry. Anal Biochem 376, 44–60. 18294950 10.1016/j.ab.2008.01.025

[mol212134-bib-0030] Hayes CA , Karlsson NG , Struwe WB , Lisacek F , Rudd PM , Packer NH and Campbell MP (2011) UniCarb‐DB: a database resource for glycomic discovery. Bioinformatics 27, 1343–1344. 21398669 10.1093/bioinformatics/btr137

[mol212134-bib-0031] Hirano K , Matsuda A , Shirai T and Furukawa K (2014) Expression of LacdiNAc groups on N‐glycans among human tumors is complex. Biomed Res Int 2014, 981627. 25003135 10.1155/2014/981627 PMC4066867

[mol212134-bib-0032] Hua S , Williams CC , Dimapasoc LM , Ro GS , Ozcan S , Miyamoto S , Lebrilla CB , An HJ and Leiserowitz GS (2013) Isomer‐specific chromatographic profiling yields highly sensitive and specific potential N‐glycan biomarkers for epithelial ovarian cancer. J Chromatogr A 1279, 58–67. 23380366 10.1016/j.chroma.2012.12.079 PMC5628020

[mol212134-bib-0033] Jacob F , Anugraham M , Pochechueva T , Tse BW , Alam S , Guertler R , Bovin NV , Fedier A , Hacker NF , Huflejt ME *et al* (2014a) The glycosphingolipid P(1) is an ovarian cancer‐associated carbohydrate antigen involved in migration. Br J Cancer 111, 1634–1645. 25167227 10.1038/bjc.2014.455 PMC4200095

[mol212134-bib-0034] Jacob F , Goldstein DR , Bovin NV , Pochechueva T , Spengler M , Caduff R , Fink D , Vuskovic MI , Huflejt ME and Heinzelmann‐Schwarz V (2012) Serum antiglycan antibody detection of nonmucinous ovarian cancers by using a printed glycan array. Int J Cancer 130, 138–146. 21351089 10.1002/ijc.26002 PMC3137667

[mol212134-bib-0035] Jacob F , Hitchins MP , Fedier A , Brennan K , Nixdorf S , Hacker NF , Ward R and Heinzelmann‐Schwarz VA (2014b) Expression of GBGT1 is epigenetically regulated by DNA methylation in ovarian cancer cells. BMC Mol Biol 15, 24. 25294702 10.1186/1471-2199-15-24 PMC4193910

[mol212134-bib-0036] Jacob F , Meier M , Caduff R , Goldstein D , Pochechueva T , Hacker N , Fink D and Heinzelmann‐Schwarz V (2011) No benefit from combining HE4 and CA125 as ovarian tumor markers in a clinical setting. Gynecol Oncol 121, 487–491. 21420727 10.1016/j.ygyno.2011.02.022

[mol212134-bib-0037] Jacob F , Nixdorf S , Hacker NF and Heinzelmann‐Schwarz VA (2014c) Reliable in vitro studies require appropriate ovarian cancer cell lines. J Ovarian Res 7, 60. 24936210 10.1186/1757-2215-7-60 PMC4058698

[mol212134-bib-0038] Jarboe E , Folkins A , Nucci MR , Kindelberger D , Drapkin R , Miron A , Lee Y and Crum CP (2008) Serous carcinogenesis in the fallopian tube: a descriptive classification. Int J Gynecol Pathol 27, 1–9. 18156967 10.1097/pgp.0b013e31814b191f

[mol212134-bib-0039] Jensen PH , Karlsson NG , Kolarich D and Packer NH (2012) Structural analysis of N‐ and O‐glycans released from glycoproteins. Nat Protoc 7, 1299–1310. 22678433 10.1038/nprot.2012.063

[mol212134-bib-0040] Jordan SJ , Green AC , Whiteman DC , Moore SP , Bain CJ , Gertig DM and Webb PM (2008) Serous ovarian, fallopian tube and primary peritoneal cancers: a comparative epidemiological analysis. International journal of cancer. Int J Cancer 122, 1598–1603. 18058817 10.1002/ijc.23287

[mol212134-bib-0041] Kawamura YI , Toyota M , Kawashima R , Hagiwara T , Suzuki H , Imai K , Shinomura Y , Tokino T , Kannagi R and Dohi T (2008) DNA hypermethylation contributes to incomplete synthesis of carbohydrate determinants in gastrointestinal cancer. Gastroenterology 135, 142–151.e143. 18485915 10.1053/j.gastro.2008.03.031

[mol212134-bib-0042] Kessler M , Fotopoulou C and Meyer T (2013) The molecular fingerprint of high grade serous ovarian cancer reflects its fallopian tube origin. Int J Mol Sci 14, 6571–6596. 23528888 10.3390/ijms14046571 PMC3645655

[mol212134-bib-0043] Kim K , Ruhaak LR , Nguyen UT , Taylor SL , Dimapasoc L , Williams C , Stroble C , Ozcan S , Miyamoto S , Lebrilla CB *et al* (2014) Evaluation of glycomic profiling as a diagnostic biomarker for epithelial ovarian cancer. Cancer Epidemiol Biomarkers Prev 23, 611–621. 24557531 10.1158/1055-9965.EPI-13-1073 PMC4230493

[mol212134-bib-0044] Kohler RS , Anugraham M , Lopez MN , Xiao C , Schoetzau A , Hettich T , Schlotterbeck G , Fedier A , Jacob F , Heinzelmann‐Schwarz V (2016) Epigenetic activation of MGAT3 and corresponding bisecting GlcNAc shortens the survival of cancer patients. Oncotarget 7, 51674–51686. 27429195 10.18632/oncotarget.10543 PMC5239506

[mol212134-bib-0045] Kurman RJ and Shih Ie M (2010) The origin and pathogenesis of epithelial ovarian cancer: a proposed unifying theory. Am J Surg Pathol 34, 433–443. 20154587 10.1097/PAS.0b013e3181cf3d79 PMC2841791

[mol212134-bib-0046] Kurman RJ and Shih Ie M (2011) Molecular pathogenesis and extraovarian origin of epithelial ovarian cancer–shifting the paradigm. Hum Pathol 42, 918–931. 21683865 10.1016/j.humpath.2011.03.003 PMC3148026

[mol212134-bib-0047] Lacy MQ , Hartmann LC , Keeney GL , Cha SC , Wieand HS , Podratz KC and Roche PC (1995) c‐erbB‐2 and p53 expression in fallopian tube carcinoma. Cancer 75, 2891–2896. 7773939 10.1002/1097-0142(19950615)75:12<2891::aid-cncr2820751216>3.0.co;2-b

[mol212134-bib-0048] Lee A , Kolarich D , Haynes PA , Jensen PH , Baker MS and Packer NH (2009) Rat liver membrane glycoproteome: enrichment by phase partitioning and glycoprotein capture. J Proteome Res 8, 770–781. 19125615 10.1021/pr800910w

[mol212134-bib-0049] Mitra I , Alley WR Jr , Goetz JA , Vasseur JA , Novotny MV and Jacobson SC (2013) Comparative profiling of N‐glycans isolated from serum samples of ovarian cancer patients and analyzed by microchip electrophoresis. J Proteome Res 12, 4490–4496. 23984816 10.1021/pr400549e PMC3846095

[mol212134-bib-0050] Nakano M , Saldanha R , Gobel A , Kavallaris M and Packer NH (2011) Identification of glycan structure alterations on cell membrane proteins in desoxyepothilone B resistant leukemia cells. Mol Cell Proteomics 10, M111.009001. 10.1074/mcp.M111.009001 PMC3226403 21859949

[mol212134-bib-0051] Nik NN , Vang R , Shih Ie M and Kurman RJ (2014) Origin and pathogenesis of pelvic (ovarian, tubal, and primary peritoneal) serous carcinoma. Annu Rev Pathol 9, 27–45. 23937438 10.1146/annurev-pathol-020712-163949

[mol212134-bib-0052] Ozols RF (2006) Challenges for chemotherapy in ovarian cancer. Ann Oncol 17(Suppl 5), v181–v187. 16807453 10.1093/annonc/mdj978

[mol212134-bib-0053] Pere H , Tapper J , Seppala M , Knuutila S and Butzow R (1998) Genomic alterations in fallopian tube carcinoma: comparison to serous uterine and ovarian carcinomas reveals similarity suggesting likeness in molecular pathogenesis. Cancer Res 58, 4274–4276. 9766651

[mol212134-bib-0054] Rader JS , Malone JP , Gross J , Gilmore P , Brooks RA , Nguyen L , Crimmins DL , Feng S , Wright JD , Taylor N *et al* (2008) A unified sample preparation protocol for proteomic and genomic profiling of cervical swabs to identify biomarkers for cervical cancer screening. Proteomics Clin Appl 2, 1658–1669. 21136816 10.1002/prca.200780146 PMC3042129

[mol212134-bib-0055] Rottmann M , Burges A , Mahner S , Anthuber C , Beck T , Grab D , Schnelzer A , Kiechle M , Mayr D , Polcher M *et al* (2017) Cancer of the ovary, fallopian tube, and peritoneum: a population‐based comparison of the prognostic factors and outcomes. J Cancer Res Clin Oncol 143, 1833–1844. 10.1007/s00432-017-2422-6 PMC11819059 28447160

[mol212134-bib-0056] Saldova R , Royle L , Radcliffe CM , Abd Hamid UM , Evans R , Arnold JN , Banks RE , Hutson R , Harvey DJ , Antrobus R *et al* (2007) Ovarian cancer is associated with changes in glycosylation in both acute‐phase proteins and IgG. Glycobiology 17, 1344–1356. 17884841 10.1093/glycob/cwm100

[mol212134-bib-0057] Saldova R , Wormald MR , Dwek RA and Rudd PM (2008) Glycosylation changes on serum glycoproteins in ovarian cancer may contribute to disease pathogenesis. Dis Markers 25, 219–232. 19126966 10.1155/2008/601583 PMC3827796

[mol212134-bib-0058] Sato T , Gotoh M , Kiyohara K , Kameyama A , Kubota T , Kikuchi N , Ishizuka Y , Iwasaki H , Togayachi A , Kudo T *et al* (2003) Molecular cloning and characterization of a novel human beta 1,4‐N‐acetylgalactosaminyltransferase, beta 4GalNAc‐T3, responsible for the synthesis of N, N’‐diacetyllactosediamine, galNAc beta 1–4GlcNAc. J Biol Chem 278, 47534–47544. 12966086 10.1074/jbc.M308857200

[mol212134-bib-0059] Schnack TH , Sorensen RD , Nedergaard L and Hogdall C (2014) Demographic clinical and prognostic characteristics of primary ovarian, peritoneal and tubal adenocarcinomas of serous histology – a prospective comparative study. Gynecol Oncol 135, 278–284. 25168689 10.1016/j.ygyno.2014.08.020

[mol212134-bib-0060] Scully RE , Young RH and Clement PB (1998) Tumors of the ovary, maldeveloped gonads, fallopian tube, and broad ligament Atlas of tumor pathology. Armed Forces Institute of Pathology, Washington, DC.

[mol212134-bib-0061] Seidman JD , Zhao P and Yemelyanova A (2011) “Primary peritoneal” high‐grade serous carcinoma is very likely metastatic from serous tubal intraepithelial carcinoma: assessing the new paradigm of ovarian and pelvic serous carcinogenesis and its implications for screening for ovarian cancer. Gynecol Oncol 120, 470–473. 21159368 10.1016/j.ygyno.2010.11.020

[mol212134-bib-0062] Sorensen RD , Schnack TH , Karlsen MA and Hogdall CK (2015) Serous ovarian, fallopian tube and primary peritoneal cancers: a common disease or separate entities – a systematic review. Gynecol Oncol 136, 571–581. 25615934 10.1016/j.ygyno.2015.01.534

[mol212134-bib-0063] Stanley P , Schachter H , Taniguchi N (2009) N‐Glycans In Essentials of glycobiology, 2nd edn (Varki A, Cummings RD, Esko JD, Freeze HH, Stanley P, Bertozzi CR, Hart GW, Etzler ME, eds), Cold Spring Harbor Laboratory Press, Cold Spring Harbor, NY. 20301239

[mol212134-bib-0064] Sun W , Grassi P , Engstrom A , Sooriyaarachchi S , Ubhayasekera W , Hreinsson J , Wanggren K , Clark GF , Dell A and Schedin‐Weiss S (2011) N‐glycans of human protein C inhibitor: tissue‐specific expression and function. PLoS One 6, e29011. 22205989 10.1371/journal.pone.0029011 PMC3242763

[mol212134-bib-0065] Tothill RW , Tinker AV , George J , Brown R , Fox SB , Lade S , Johnson DS , Trivett MK , Etemadmoghadam D , Locandro B *et al* (2008) Novel molecular subtypes of serous and endometrioid ovarian cancer linked to clinical outcome. Clin Cancer Res 14, 5198–5208. 18698038 10.1158/1078-0432.CCR-08-0196

[mol212134-bib-0066] Uphoff CC and Drexler HG (2005) Detection of mycoplasma contaminations. Methods Mol Biol 290, 13–23. 15361652 10.1385/1-59259-838-2:013

[mol212134-bib-0067] Varki A , Cummings RD , Aebi M , Packer NH , Seeberger PH , Esko JD , Stanley P , Hart G , Darvill A , Kinoshita T *et al* (2015) Symbol nomenclature for graphical representations of glycans. Glycobiology 25, 1323–1324. 26543186 10.1093/glycob/cwv091 PMC4643639

[mol212134-bib-0068] Vaughan S , Coward JI , Bast RC Jr , Berchuck A , Berek JS , Brenton JD , Coukos G , Crum CC , Drapkin R , Etemadmoghadam D *et al* (2011) Rethinking ovarian cancer: recommendations for improving outcomes. Nat Rev Cancer 11, 719–725. 21941283 10.1038/nrc3144 PMC3380637

[mol212134-bib-0069] West MB , Segu ZM , Feasley CL , Kang P , Klouckova I , Li C , Novotny MV , West CM , Mechref Y and Hanigan MH (2010) Analysis of site‐specific glycosylation of renal and hepatic gamma‐glutamyl transpeptidase from normal human tissue. J Biol Chem 285, 29511–29524. 20622017 10.1074/jbc.M110.145938 PMC2937983

