# Supplementary material for: Tissue glycomics distinguish tumour sites in women with advanced serous adenocarcinoma
Source: Mol Oncol. 2017 Sep 29;11(11):1595–615. doi: 10.1002/1878-0261.12134 (PMC5663998; doi:10.1002/1878-0261.12134)
Supplement: Supplementary file 1 — Fig. S1. Relative quantitation of abundances of sialylation levels (mono‐, di‐ and tri‐) and branching patterns of N‐ and O‐glycans. Fig. S2. Representative MS2 fragment ion spectra depicting the diagnostic ions of bisecting GlcNAc type N‐glycans in serous cancers. Fig. S3. Representative extracted ion chromatograms (EIC) of glycan masses bearing terminal LacdiNAc (GalNAc‐GlcNAc) at m/z 913.92− and sialylated LacdiNAc (Neu5Ac‐GalNAc‐GlcNAc) at m/z 1205.02− serous ovarian and peritoneal cancers. Table S1. Source and clinico‐pathological information of serous cancer specimens. Table S2. Proposed N‐ and O‐glycan structures detected on the membrane proteins of serous cancers derived from the ovary and peritoneum. Table S3. QPCR parameters providing the standard curve parameters for each primer pair on 8 targeted genes. Table S4. QPCR parameters providing the standard curve parameters for each primer pair on 8 targeted genes. [file MOL2-11-1595-s001.docx]

**Supplementary Table 1**


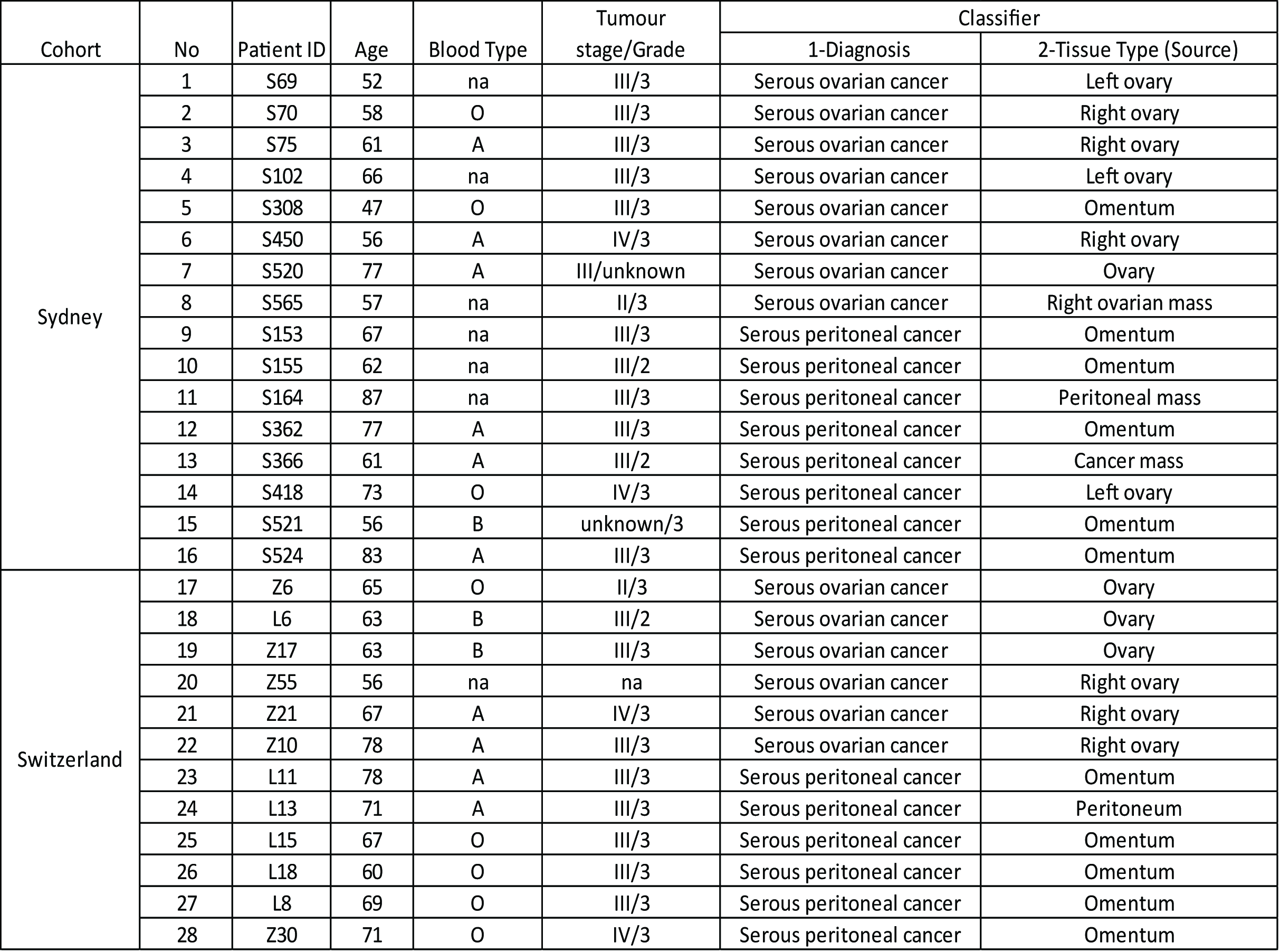


**Supplementary Table 2**

**
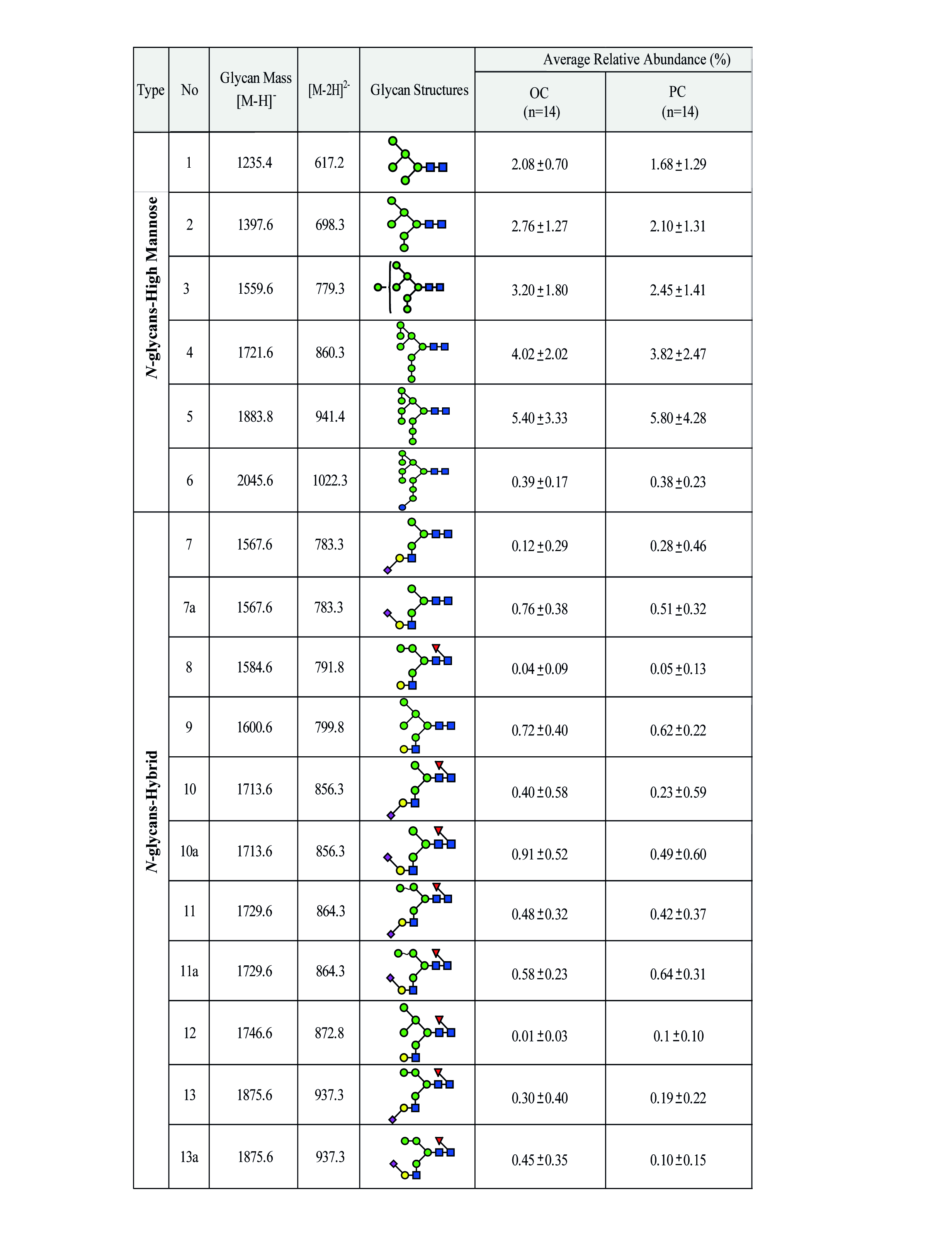
**

**
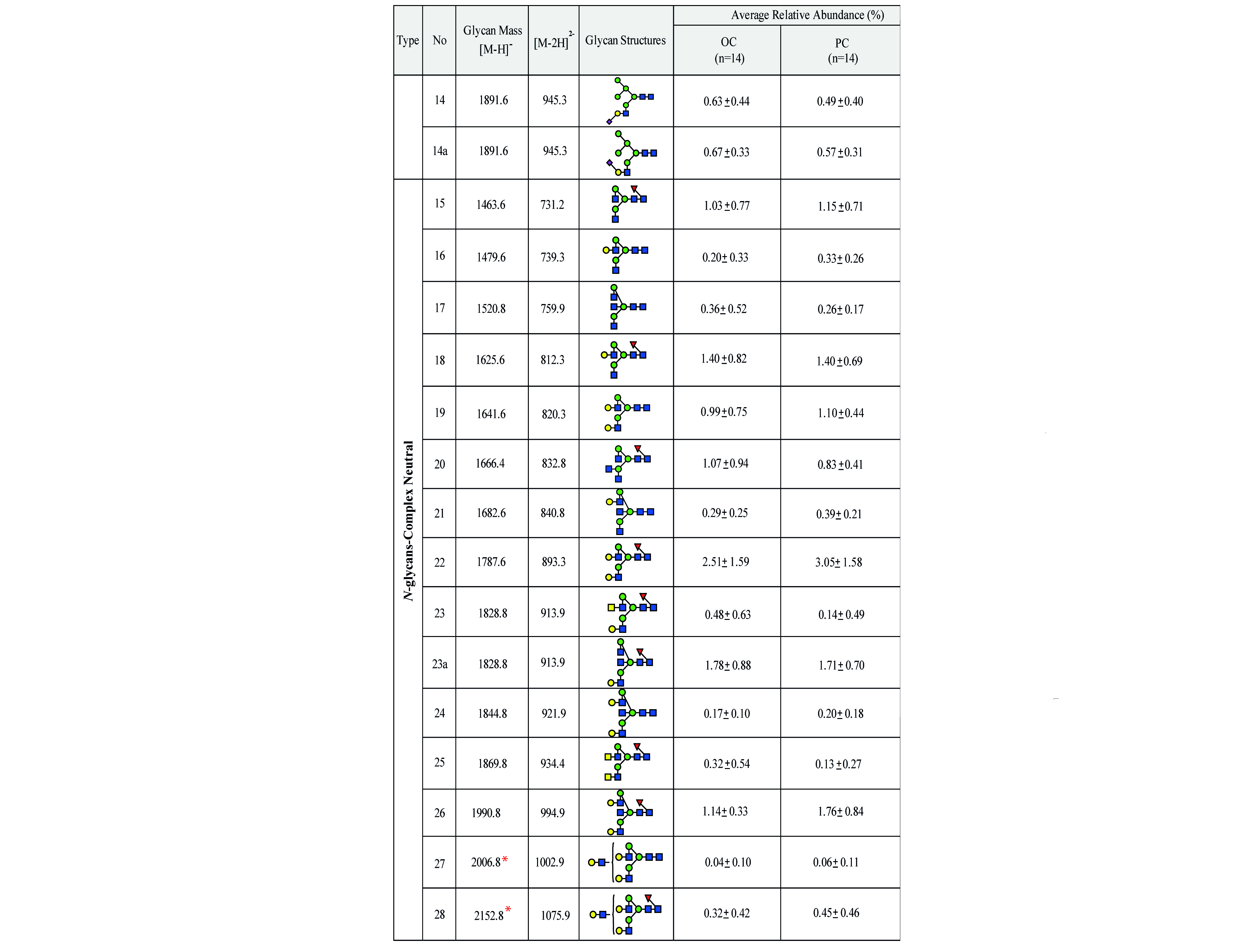
**

**
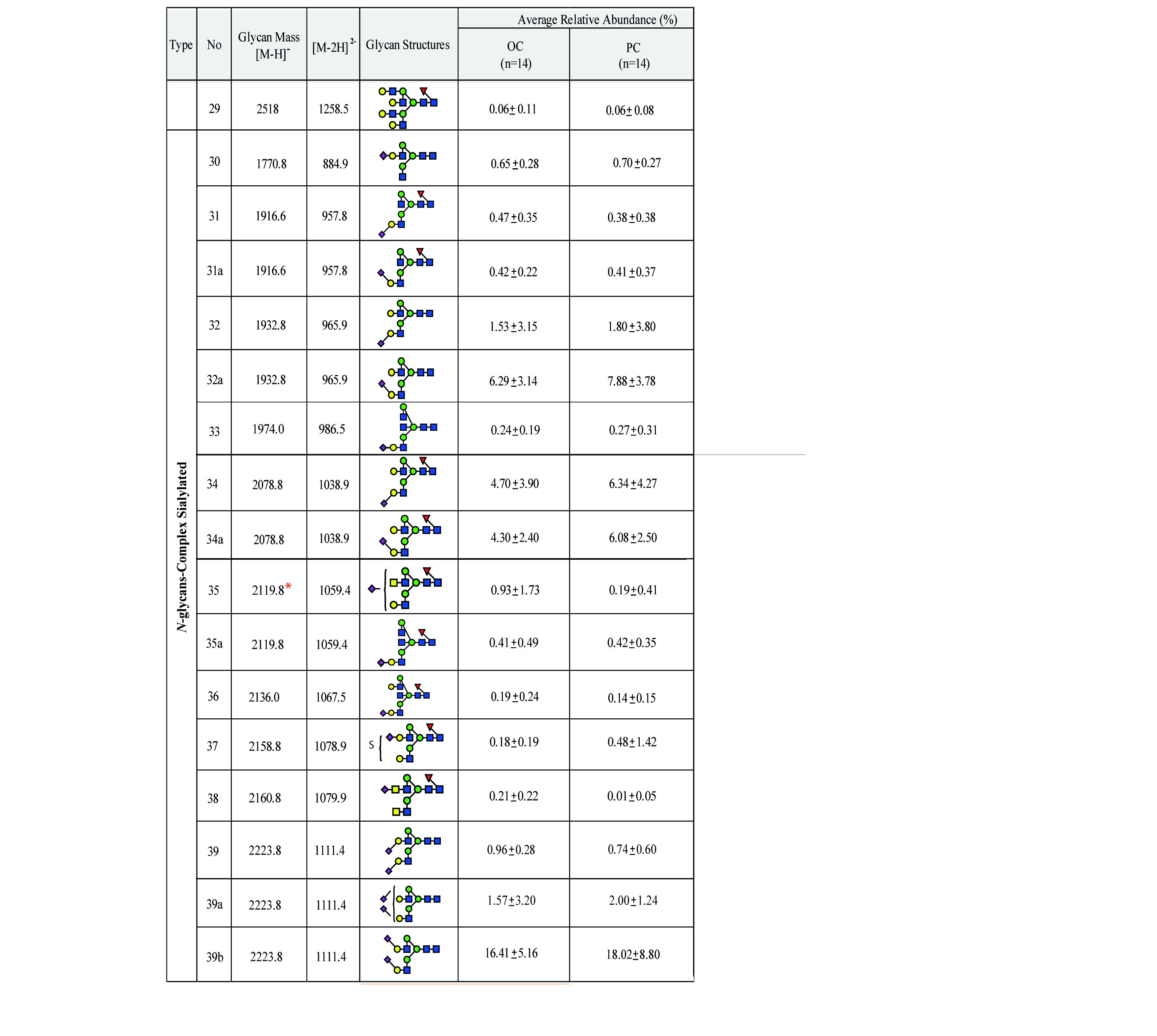
**

**
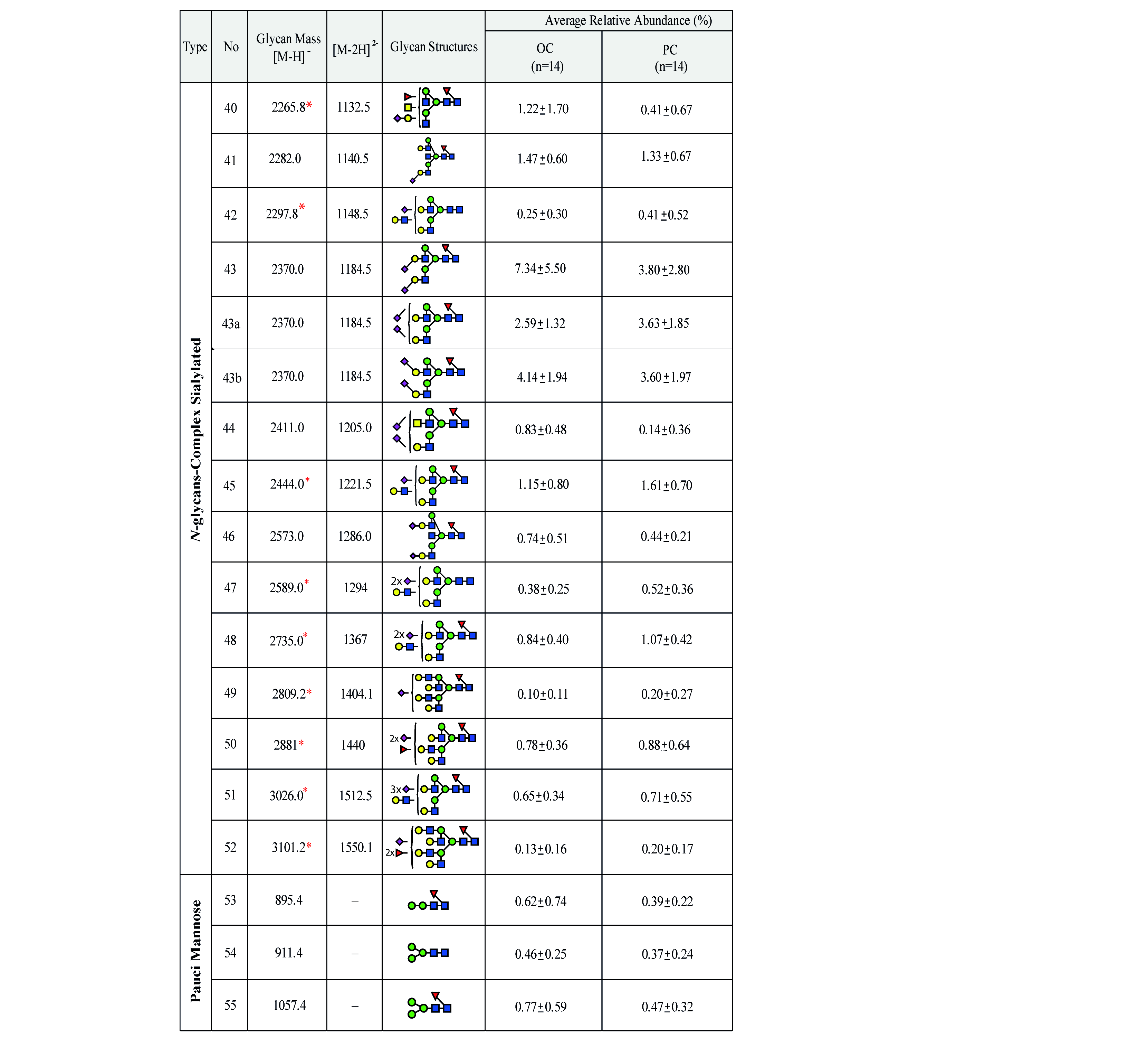
**

**
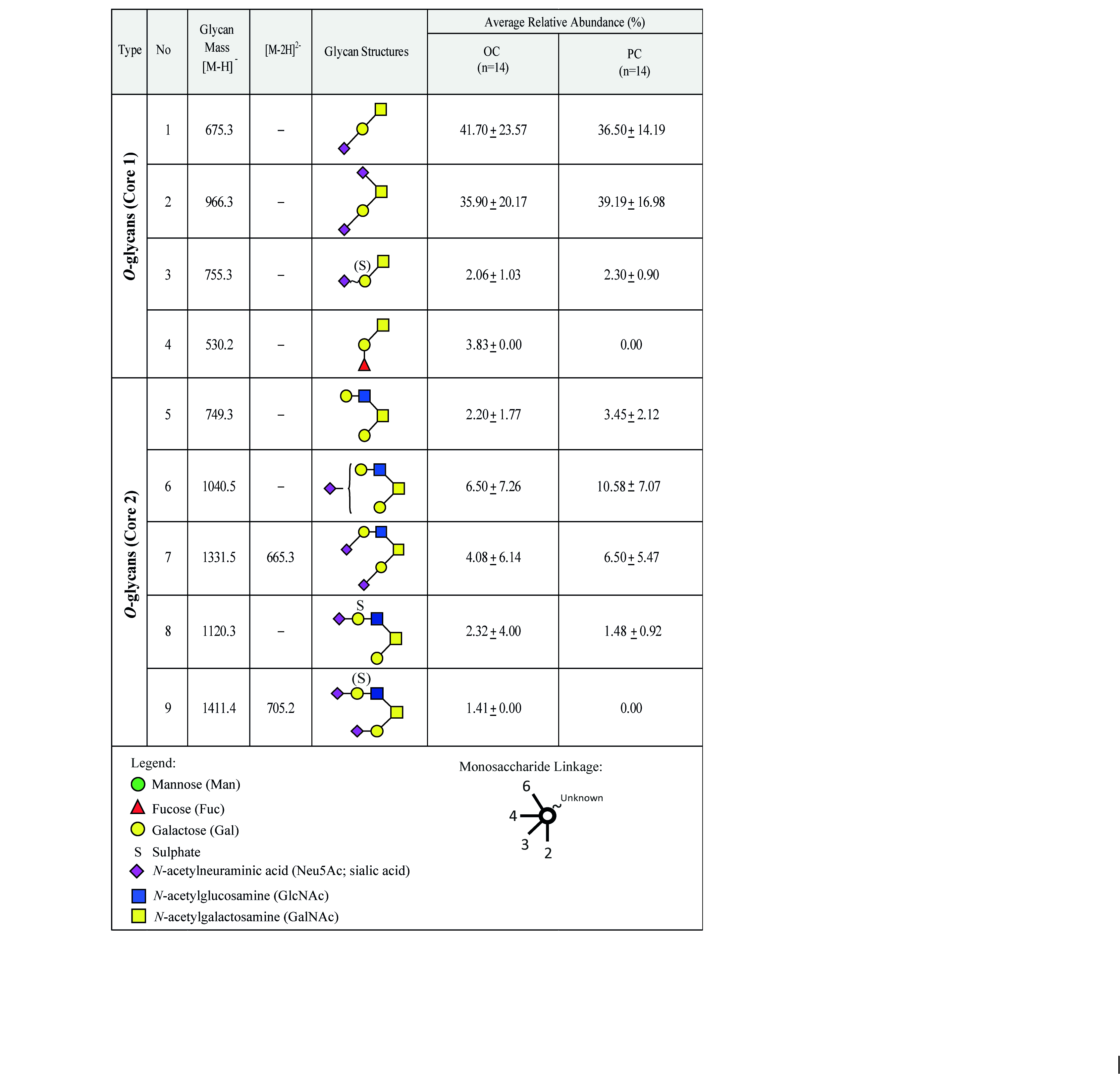
**

**Supplementary Table 3**

| **Gene Name** | **Abbr.** | **Forward Primer 5’-3’** | **Reverse Primer** | **Amplicon size in bp** | **Intron spanning** | **Ref.** | **note** |
| --- | --- | --- | --- | --- | --- | --- | --- |
| **Heat shock protein 90kDa alpha (cytoslic)** | *HSPCB* | TCTGGGTATCGGAAAGCAAGCC | GTGCACTTCCTCAGGCATCTTG | 80 | Yes | [^1^](#_ENREF_1) | reference |
| **Tyrosine 3-monooxygenase/ tryptophan 5-monooxygenase activation protein, zeta polypetide** | *YWHAZ* | ACTTTTGGTACATTGTGGCTTCAA | CCGCCAGGACAAACCAGTAT | 94 | NA | [^2^](#_ENREF_2) | reference |
| **Succinate dehydrogenase complex, subunit A** | *SDHA* | TGGGAACAAGAGGGCATCTG | CCACCACTGCATCAAATTCATG | 86 | Yes | [^2^](#_ENREF_2) | reference |
| **Beta-1,4-N-acetylgalactos-aminyltransferase 3** | *B4GALNT3* | TGGCATAGACCTCGTGAAGGAC | ACAGTGCTTCCGAATGGCATC | 100 | Yes | QPPD | glyco |
| **Beta-1,4-N-acetylgalactos-aminyltransferase 4** | *B4GALNT4* | AGAAGATCCGTAAGCAGATGAAGC | TGGTCAGCTTCTCACCATCTCG | 150 | Yes | QPPD | glyco |
| **Beta-galactosamide-alpha-2,3-sialyltransferase 4** | *ST3GAL4* | CCCTGGCTGCTCTTATGGAG | AGAGAGGGGGTGCCACAG | 103 | Yes | QPPD | glyco |
| **Beta-galactosamide-alpha-2,3-sialyltransferase 5** | *ST3GAL5* | CCTCATTAGTATGCGGACGAAGGC | AGGTGTACTCACTTGGCATTGCTC | 113 | Yes | QPPD | glyco |
| **Beta-galactoside alpha-2,6-sialyltransferase 2** | *ST6GAL2* | TTCCTTGGGCGAGGAAATAGATTC | TCATAACCACGTGTAGGAGCAGAG | 72 | Yes | QPPD | glyco |

**Supplementary Table 4**

| **Gene** | **Uniprot** | **cell line** | **slope** | **Efficiency** | **R^2^** | **dilution range** |
| --- | --- | --- | --- | --- | --- | --- |
| ***HSPCB*** | P08238 | SKOV3 | -3,250 | 103.1 | 0.998 | 1pg – 100ng |
| ***YWHAZ*** | P63104 | SKOV3 | -3.294 | 101.2 | 0.998 | 1pg – 100ng |
| ***SDHA*** | P31040 | SKOV3 | -3.194 | 105.6 | 0.994 | 1pg – 100ng |
| ***B4GALNT3*** | Q6L9W6 | OVCAR3 | -3.313 | 100.4 | 0.998 | 500pg – 50ng |
| ***B4GALNT4*** | Q76KP1 | EFO27 | -3.198 | 105.4 | 0.967 | 5pg – 50ng |
| ***ST3GAL4*** | Q11206 | IGROV1 | -3.474 | 94.0 | 0.995 | 5pg – 50ng |
| ***ST3GAL5*** | Q9UNP4 | EFO27 | -3.540 | 91.6 | 0.949 | 5pg – 50ng |
| ***ST6GAL2*** | Q96JF0 | OVCAR3 | -3.699 | 86.4 | 0.997 | 500pg – 50ng |

**Supplementary Figure 1**

**
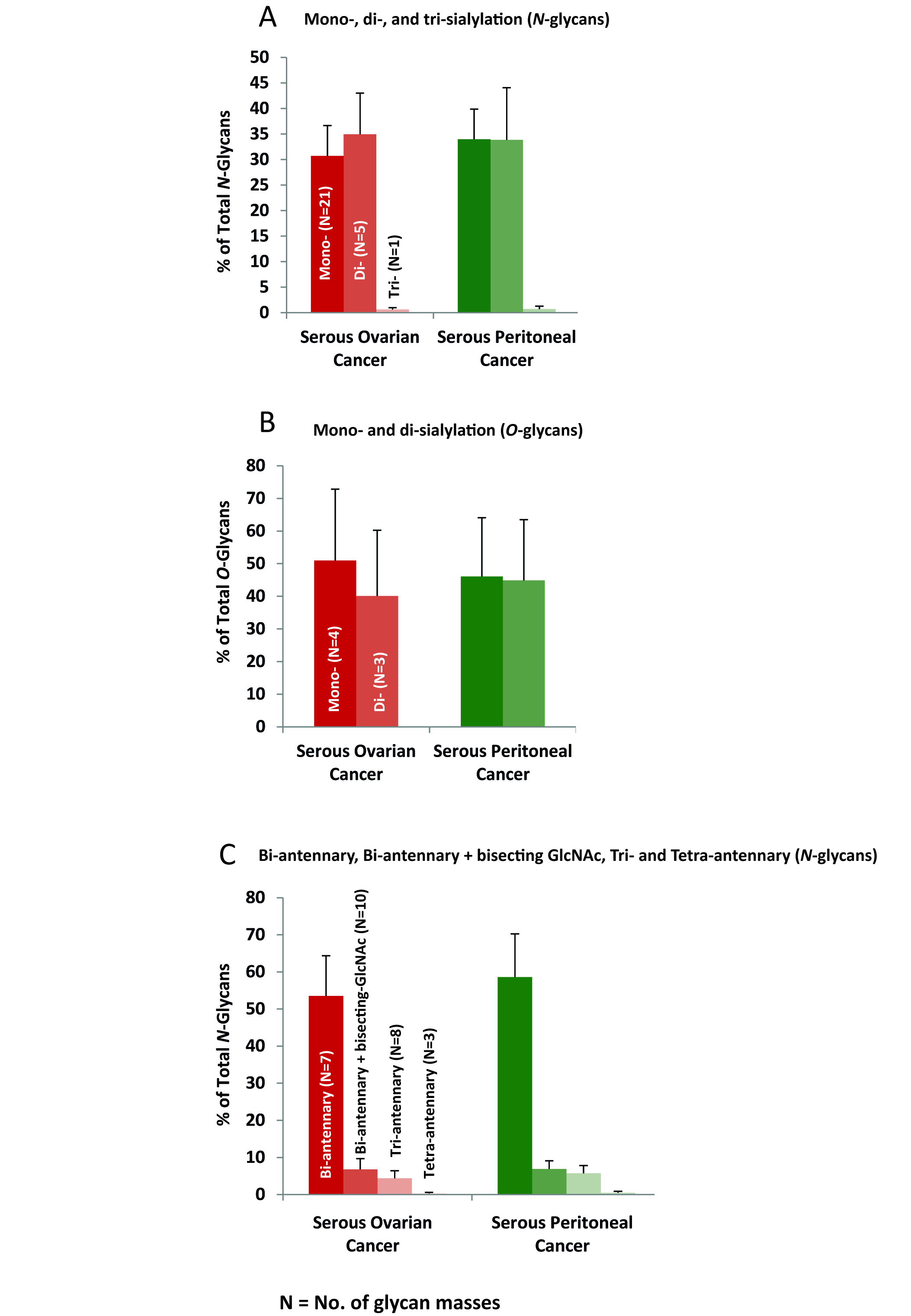
**

**Supplementary Figure 2**

**
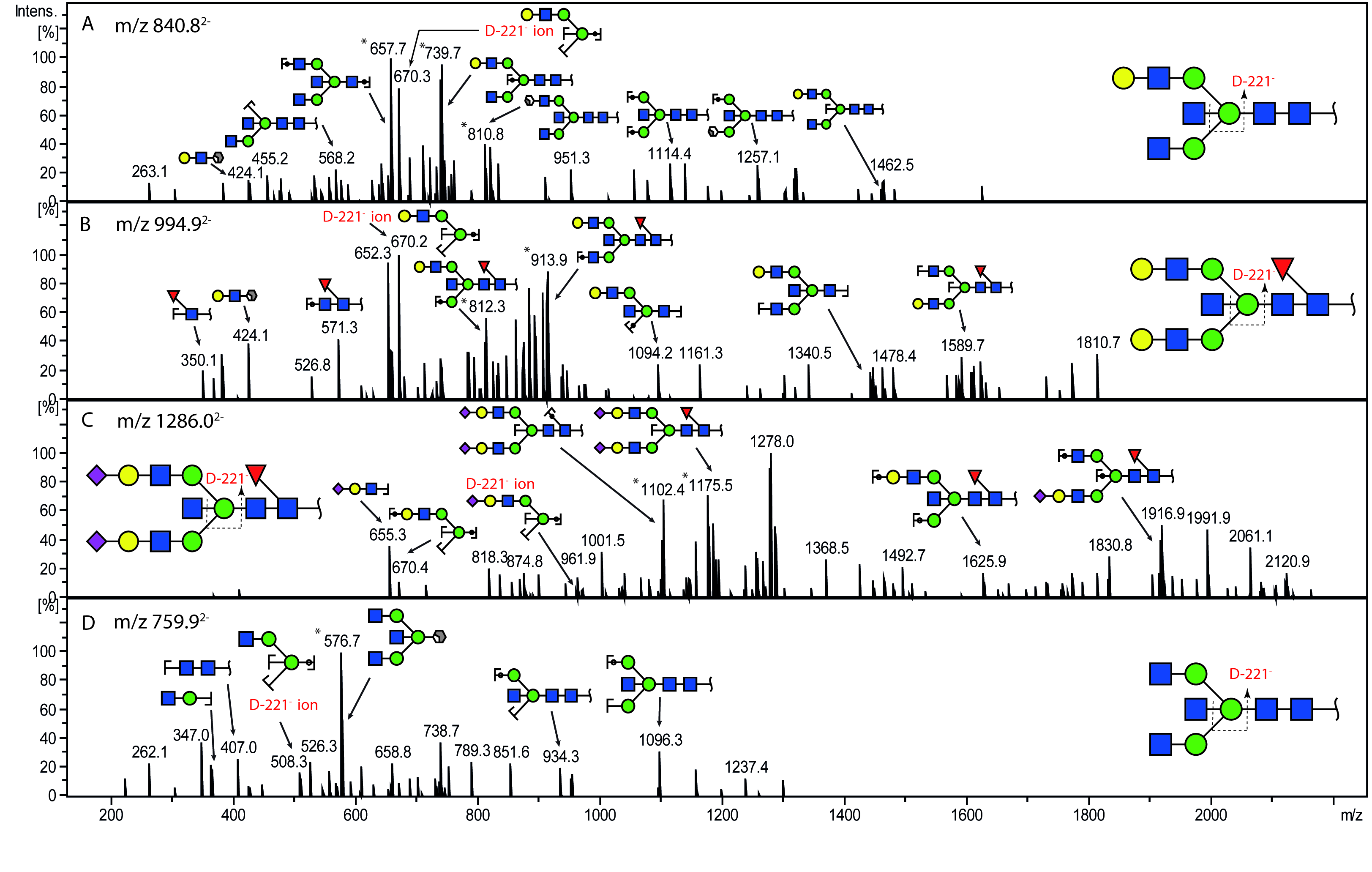
**

**Supplementary Figure 3**

**
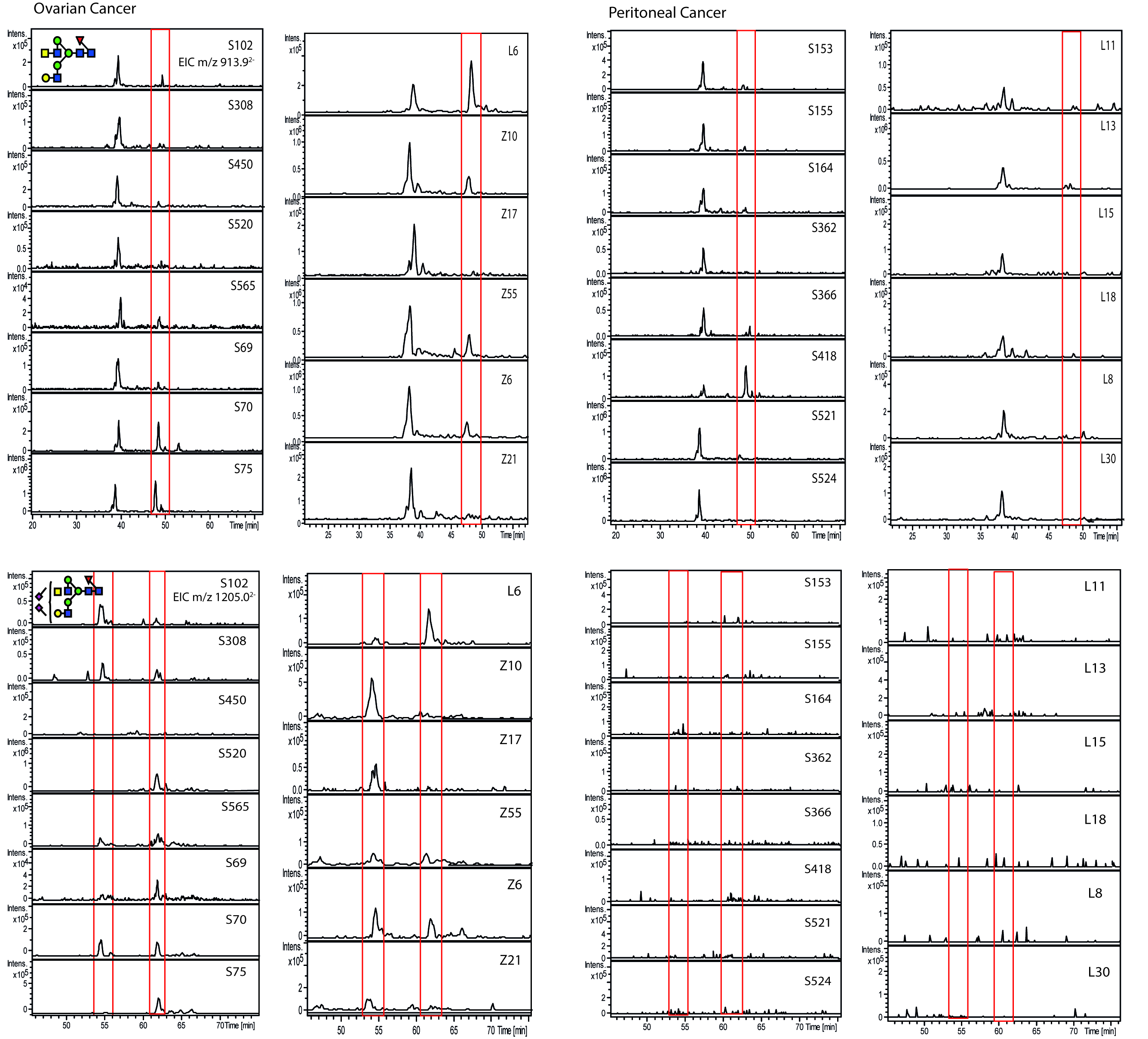
**

**References**

1. Arvidsson S, Kwasniewski M, Riano-Pachon DM, Mueller-Roeber B. QuantPrime--a flexible tool for reliable high-throughput primer design for quantitative PCR. *BMC bioinformatics* 2008; **9**: 465.

2. Vandesompele J, De Preter K, Pattyn F, et al. Accurate normalization of real-time quantitative RT-PCR data by geometric averaging of multiple internal control genes. *Genome biology* 2002; **3**(7): RESEARCH0034.
